# A New Test for Irony Detection: The Influence of Schizotypal, Borderline, and Autistic Personality Traits

**DOI:** 10.3389/fpsyt.2019.00028

**Published:** 2019-02-14

**Authors:** Carolin Kieckhäfer, Anne K. Felsenheimer, Alexander Michael Rapp

**Affiliations:** ^1^Department of Psychiatry and Psychotherapy, University of Tuebingen, Tuebingen, Germany; ^2^Department of Psychiatry, LVR-Hospital Düsseldorf, Heinrich-Heine-University Düsseldorf, Düsseldorf, Germany; ^3^Fliedner Klinik Stuttgart, Stuttgart, Germany

**Keywords:** irony comprehension, sarcasm, social cognition, figurative language, schizophrenia, praise

## Abstract

Irony has repeatedly been suggested as a language based social cognition task. It has been argued to show specific variances in psychiatric disorders and healthy adults with certain personality traits. Above that, irony comprehension is based on a complex interplay of the informational context, the relationship of the conversational partners, and the personality of the recipient. The present study developed a video-based German language test for a systematic examination of irony detection accuracy (Tuerony). The test includes (i) a stereotypical conversation partner (doctor, actor) in (ii) different perspectives (direct interaction, neutral observer) and (iii) a bilateral chat history on a conventional messenger service interface with ironic criticism, ironic praise, literal criticism, and literal praise. Based on the continuous approach of psychiatric symptoms, schizotypal, borderline, and autistic personality traits were associated with irony detection accuracy in a healthy sample. Given the often reported role of mentalization in irony detection, these associations were also investigated. First, a broad variance of irony comprehension in our healthy sample could be shown. Second, schizotypal and borderline, but not autistic traits were significantly negatively associated with irony detection accuracy. Finally, in the current healthy sample, neither variation of the conversational context nor mentalization characteristics were significantly associated with performance beyond personality traits. The current results therefore highlight two aspects for future research in irony comprehension: the importance of ecological valid tests and the role of the individual personality of the recipient.

## Introduction

Irony comprehension has recently gained a remarkable increase in importance as a subtle pragmatic task for social functioning. Impairments of verbal irony comprehension have been known for a long time in clinical populations, such as persons with autism (1) and schizophrenia (2), and for a short time in non-clinical populations with high characteristics of certain personality traits. There has been a growing body of research highlighting the dimensional instead of categorical character of psychiatric personality traits and corresponding cognitive impairments in healthy adults (3). For example, there is reason to assume that autistic personality traits represent a continuum with manifest Asperger syndrome (4). Same suggestions have been made for personality disorders (4, 5), showing associations between a dimensional approach and psychosocial symptoms in borderline traits (6), and schizotypy (7, 8). Assuming this continuity of personality traits, the reduced performance of irony comprehension in clinical populations raises the question on whether these deficits are related to irony comprehension accuracy in healthy adults as well. Nonetheless, in the field of irony research the dimensional approach has rarely been investigated (9). Langdon and Coltheart (10) showed that healthy adults with high schizotypal trait characteristics performed significantly worse in verbal irony comprehension. This relation between psychometric schizotypy and irony comprehension in a non-clinical population as observed in neuropsychological tests could not be shown by Jahshan and Sergi (11). On the neurobiological level, Rapp et al. (12) found a significant decrease of activation in the middle temporal gyrus during irony comprehension in subjects with high schizotypal personality characteristics.

As a possible reason for the heterogeneous results, this research draws further attention to a natural variance in irony comprehension. Apart from the symptom-related impairment of irony comprehension in clinical populations, differences in accuracy in detecting ironic remarks have also been found in healthy individuals, although to a much lesser degree (13). A possible suggestion implies a time-stable cognitive accuracy in detecting irony. Whereas, one individual may robustly show an almost perfect performance in detecting ironic intentions, another individual, despite having no manifest impairment in language comprehension, and use in general, may more often fail. Such an ability of verbal irony comprehension would interact with known factors that influence verbal irony comprehension, for example, the availability of contextual information. Assuming that an individual has a given “irony detection accuracy” would imply several things: the interindividual differences in accuracy may represent valuable information, not just noise. The individual irony detection accuracy may modulate comprehension performance by affecting established factors of the comprehension process. For instance, the difficulty of the stimulus or the quality of context information may be processed diversely according to the individual performance. Taking a closer look at the concrete process of irony comprehension, encoding the meaning of ironic utterances generally demands a set of different linguistic features and social skills for irony comprehension: world knowledge/common sense (14, 15), meta-representation (16), social context information (17, 18), cultural information (19, 20), familiarity (21), and prosody (22, 23).

In clinical contexts, irony is usually defined as an opposition between the literal and non-literal meaning of a given statement (24). Thus, the social cognition basis for irony comprehension is building representations of one's own and others' mental states, which is a prerequisite for irony use and comprehension, necessary to encode (speaker) and decode (listener) the opposite meaning. The abstract concept for the representations remains manifold and varies from the terms theory of mind [ToM; (25)], meta-representation (26), mind reading (27), and perspective-taking to mentalization (28). All of these terms comprise aspects of social cognition (29) and are generally used to describe the human ability to have thoughts about thoughts and to make inferences on the thoughts of others (14, 30).

In line with this, ToM as an underlying mechanism is a shared deficit in autism and schizophrenia (31–33) and has been shown to account for their specific impairments in irony (10, 34). However, ToM deficits do not seem to be restricted to autism and schizophrenia. Quite the contrary, there are numerous other psychiatric [unipolar depression: (35); bipolar affective disorder: (36); antisocial personality disorder: (37)] and somatic disorders [Parkinson's disease: (38); frontotemporal dementia: (39, 40)] with broadly varying types of representational deficits leading to distinct symptoms. Along with this, there is growing research on ToM differences associated with borderline personality disorder (41, 42).

Indeed, borderline personality traits may represent a candidate trait that could theoretically be related to irony detection accuracy. There is extensive rumor and anecdotes among clinicians that borderline personality patients exhibit a misinterpretation of ironic intentions. Moreover, there is some evidence of impaired irony comprehension in borderline personality disorder (43). This highlights not only the necessity to elucidate whether other personality traits may also be associated with irony detection accuracy in a similar or even more robust way (44), but also which role ToM plays beyond or within those personality traits. Investigating these associations in a healthy sample might help to elucidate possible explanations for the natural variance in irony comprehension in our everyday life. In fact, and in line with the assumption of a continuous model, healthy adolescents with borderline traits seem to differ in their ToM abilities (45), too. Moreover, autistic personality traits interrelate with other measures of social cognition in a comparable size schizotypal traits do (46, 47). Nevertheless, to the best of our knowledge, no previous work has investigated the relationship to irony comprehension in non-clinical individuals with only subthreshold autistic and borderline traits and research on schizotypal personality traits in healthy adults remain limited.

The importance of irony comprehension in everyday life and the complex cognitive requirements underline the importance of high ecological validity in tests of irony detection (48–51). This high ecological validity empowers irony comprehension tests to differentiate better than other social cognition and empathy measures between schizophrenia and other diagnoses (52). Beyond that, training in irony comprehension has been repeatedly suggested to be a target for social cognition training in these patients (48, 49).

In the English language, the Awareness of Social Inference Test [TASIT; (53)] is by far the most applied irony comprehension paradigm with high ecological validity (2). However, although the TASIT has already been translated into other languages (54, 55), it is not yet available in German. The video-based TASIT includes information on facial expression and prosody from the speaker such that adding prosody-free tests without facial expressions seems reasonable to show that an assumed deficit in a clinical population is not explained solely by these factors. Furthermore, an eligible, more profound knowledge of the distinct mechanisms in irony comprehension is of special interest because the frequencies with which ironic remarks are misunderstood are dramatic even in healthy subjects (20, 56). It is therefore consequent to evaluate factors associated with irony comprehension performance in healthy subjects.

One of those often suggested factors is the social and cultural information about the ironic speaker (18, 57, 58). Pexman and Olineck (18) showed that healthy participants regard some occupations, such as actors and comedians, as more sarcastic than others, such as clergymen and physicians. However, the results on the influence of stereotypical occupations in clinical populations are inconsistent. A study by Champagne-Lavau and Charest (59) used the suggested occupations in a study with schizophrenia patients and matched healthy participants. In both groups, the irony detection depended significantly on the stereotype of the speaker, with greater performances observed for “sarcastic occupations” (e.g., actresses) than for “non-sarcastic occupations” (e.g., scientists and veterinarians). Castelli et al. (60) provided support for this view when they showed that general knowledge about stereotypes seemed to be preserved in patients with schizophrenia, enabling the same perception of stereotypical occupations in schizophrenic patients as that in healthy adults. Zalla et al. (61) applied an analogous study design in patients with autism spectrum disorders and in healthy controls. In contrast to previous studies, significantly improved irony detection for speakers with a sarcastic occupation was found only in healthy controls. Based on these findings, Zalla et al. (61) argued that although occupational stereotypes are perceived in patients with autistic spectrum disorders, they are not as much integrated in the pragmatic process as in healthy controls.

The second, more direct influence of context on the irony comprehension process might be the relationship the individual has to the speaker of ironic utterances. Although in everyday life, irony rather occurs when someone is directly talking to you, most paradigms investigate stimuli where two others are talking to each other. More precisely, the recipient of irony in these tests is always someone else and never the participant. However, these situations differ immensely in the degree of self-involvement of the participant. This is because irony, and conversational turns in general, toward a protagonist may be interpreted differently from irony among other protagonists, and this effect may be even much larger in clinical populations. Parallel to our investigation, the importance of self-involvement and perspective-shifting in irony processing has been recently foregrounded by several authors (62–65). Unfortunately, only a limited number of studies have investigated this topic. Those that did, usually interchanged direct involvement with the amount of perspective-taking. For example, Deliens et al. (65) used a design for sarcasm detection, which involved the participant indirectly by giving more information to the participant than to the addressee in given scenarios. Under this asymmetry of information, the participants had significant deficits in sarcasm detection compared with scenarios with shared perspectives. Deliens et al. (65) argued that these differences were based on the additional cost for the necessary shift of perspectives. However, the setup seemed to be more of an adult version of the Sally-Ann scenario (66), in which the participant was not personally addressed. Instead, self-involvement was implemented only in the amount of information given. In a functional magnetic resonance imaging (fMRI) study by Akimoto et al. (63), Japanese participants were instructed to read stories with a first-person view and then to decide on whether utterances directed to the character were ironic or literal. However, the authors focused on neural mapping of relevant brain regions for irony comprehension and did not control for self-involvement with third-person view stories; thus, such influence was not thoroughly investigated.

In the present work, to attain particular personality trait–weighted differences and to avoid overly simple requirements for irony understanding in clinical and non-clinical populations, a new test for irony comprehension under various conditions is carried out, which takes previous findings into consideration. In the first step, the Tuebingen Test of Irony Detection Accuracy (tuerony) is introduced and evaluated, which combines a set of the most relevant conditions: speakers' occupational stereotypes, a variation of self-involvement of the addressee and the content of the remark, being either critical or praising and comparing ironic with literal statements. In the second step, the tuerony is used to systematically investigate the natural variance of irony detection accuracy in a healthy population. Finally, the underlying mechanisms of the comprehension process, in relation to schizotypal, borderline and autistic traits are examined according a dimensional model in a healthy sample and related to mentalization and the relevant context conditions of the test.

## Materials and Methods

### Participants

Ninety-six (59 females, 61.5%) healthy and unmedicated adults with no history of psychiatric disorders took part in the current study. All participants were native German speakers. Mental and noteworthy physical disorders served as exclusion criteria. Recruitment was done through social platforms and advertisement at the University of Tuebingen, Germany. Table 1 shows the personality and demographic characteristics of the study sample. Within this preliminary evaluation we mainly focused on possible influences on the detection process. Thus, the sample size was chosen in order to test these influences instead of fulfilling the full criteria for test construction. Because the main analysis comprised 4 subscales and 3 possible predictors, we oriented on the commonly suggested ratio of observed variables and cases 1:10, resulting in a minimum of 70 participants.

**Table 1:** Means (M), standard deviations (SD) and range (Min, Max) of personality and demographic characteristics of the study sample (*N* = 96).

	**M**	**SD**	**Min**	**Max**
Age	26.43	7.52	18	55
General Intelligence	30.39	3.60	20	36
Gender	59 females/37 males			
Share of students	76			
**SPQ**				
Total	14.67	9.97	0	40
Cogntive perceptual	9.97	7.04	0	30
Interpersonal	6.06	5.33	0	25
Referential thinking	2.21	2.00	0	8
Social anxiety	1.83	1.90	0	6
Magical ideation	0.81	1.25	0	6
Unusual perceptual experiences	1.30	1.52	0	8
Odd or eccentric behavior	1.45	1.60	0	7
No close friends	1.24	1.65	0	8
Odd speech	2.83	2.33	0	9
Constricted affect	1.63	1.76	0	7
Suspiciousness	1.36	1.38	0	5
**AQ**				
Total	15.97	5.57	4	33
Social	2.14	1.99	0	8
Attention switching	4.35	1.82	1	9
Attention to detail	4.57	2.06	1	10
Communication	2.08	1.79	0	8
Imagination	2.82	2.12	0	10
**BSL-23**				
Total	11.02	12.02	0	67
Dysfunctional behavior	1.20	1.66	0	7
**SEE**				
Congruence	22.65	4.37	9	30
Overwhelming emotions	17.99	5.77	7	31
Lack of emotions	11.33	3.85	5	22
Symbolization by bodily experience	23.77	6.34	10	38
Symbolization by imagination	14.71	5.45	6	27
Emotion Regulation	12.36	2.83	6	20
Self-Control	20.61	3.81	11	28
**IRI**				
Total	44.20	5.46	32	58
Fantasy	13.80	3.02	7	20
Empathic concern	14.84	2.30	10	20
Perspective taking	15.55	2.65	9	20
Personal distress	11.01	2.98	5	20
**STHI**				
Cheerfulness	33.21	5.43	15	40
Seriousness	26.98	4.70	15	37
Bad mood	19.25	6.15	10	35
**TOSCA**				
Shame	31.94	7.59	14	46
Guilt	45.25	4.15	35	53
Externlization	23.91	6.09	11	37
Detached	32.90	5.79	18	47
Edinburgh Handedness Inventory	91 right/5 left			

*SPQ, Schizotypal Personality Questionnaire; AQ, Autism Spectrum Quotient; BSL-23, Borderline Symptom List 23; SEE, Subjective Experience of Emotions Scale; IRI, Interpersonal Reactivity Index; STHI, State-Trait-Heiterkeits-Inventory; TOSCA, Test of Self-Conscious Affect*.

### Materials

#### Tuebingen Test of Irony Detection Accuracy (Tuerony)

**Figure 1:**
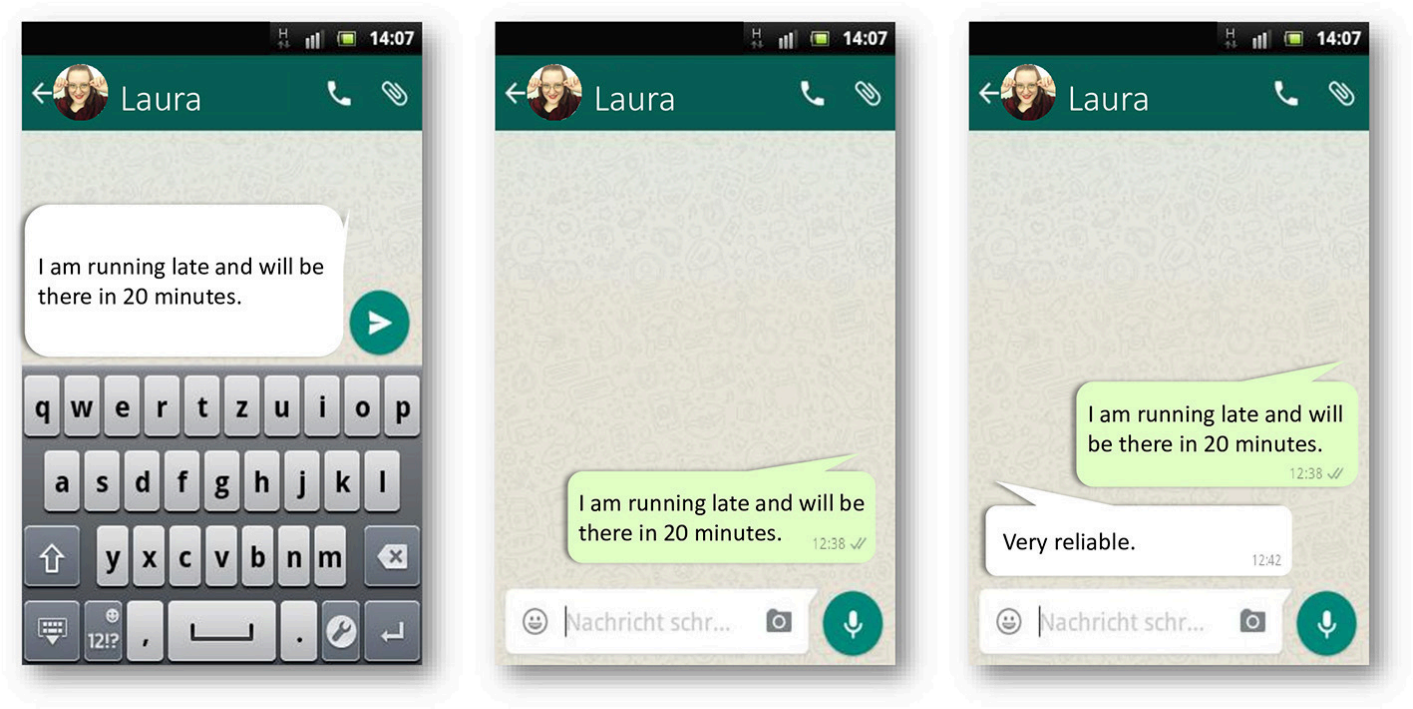
Items were presented in a common messenger service interface.

The Tuebingen Test of Irony Detection Accuracy (tuerony) is a social cognition and social language comprehension paradigm for use in clinical and non-clinical populations. The tuerony is computerbased and intended to be performed under laboratory conditions. In this study, it was implemented via the SoSciSurvey online platform (67). The test consists of videotaped context stories and written ironic, literal, critical, and praising remarks. The stimuli are embedded in a smartphone messenger service interface currently operated by many individuals (see Figure 1). The intended time for tuerony ranges from 10 to 20 min. However, no time limit is given.

Every test run begins with a written instruction, followed by a short video sequence to establish a coherent context for subsequent item presentation (Figure 1). There are four possible videos for the test, all sharing one narrative, in which a fictitious character moved into town and meets another protagonist in a café. These video sequences vary in two ways (context condition). First, the character is either a physician or an actor (variation of stereotype). Both characters were either performed by a woman or a man, who turned out to be both actors as well as physicians in their every-day life, facilitating an authentic depiction of each role. For all figures and videos written informed consent was obtained for testing and publication from both actors and their names were changed for the videos, instructions and depicted conversations. Second, the video depicts this character talking to a neutral other person or directly toward the participant (variation of self-involvement). In the latter, participants are instructed to picture themselves that they are meeting the new character in the café. This variation of self-involvement was established through camera angle techniques with a neutral shot for the observing condition and a point-of-view shot for direct interaction with the participant. To guarantee differing degrees of self-involvement, actors were instructed to keep eye contact only with each other in videos for neutral observation and look directly and frontal into the camera in videos for the direct interaction. An example of an original video instruction is given in Supplementary Video 1.

**Figure 2:**
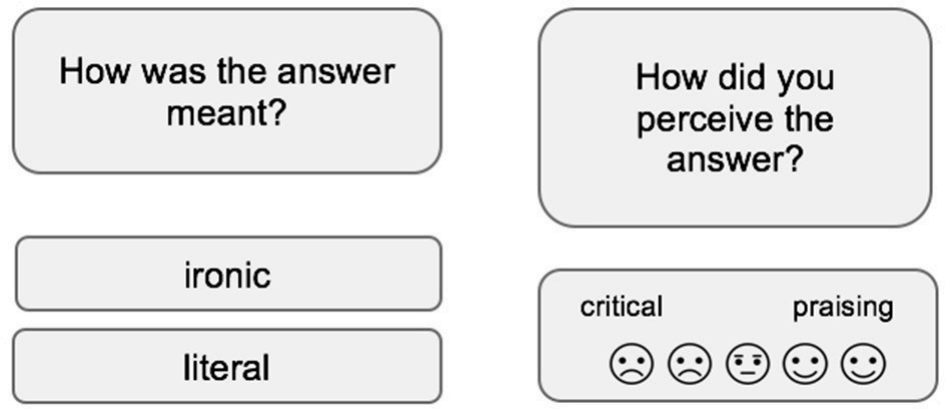
Items were evaluated first on a dichotomous scale (ironic vs. literal) and second on a five-point smiley-based Likert scale (critical to praising).

After the video, participants get to read 20 conversational turns in the format of short text messages. While they are presented as conversations *among* a character and a neutral other in the observation condition, participants directly exchange predefined messages *with* the introduced character in the interaction condition. All dialogues were structured in two parts, providing information on an everyday situation of one speaker at first (context sentence) and a direct verbal reaction on that by another (target sentence). The items themselves are to be read, so that no prosodic or face expression information is given. Above that, no further paraverbal hints, such as emoticons, are given as indicators for irony. Thus, correct answers could only be hints for correct pragmatic and intentional comprehension simply based on content of the messages. Consequently, each answer of the introduced character must be evaluated by the study participant on a dichotomous scale on literality (ironic vs. literal). Afterwards, it is evaluated on a five-point smiley-based Likert scale on perceived intention (critical vs. praising) (see Figure 2).

In the final version, a participant completed two test versions, with crossed conditions of context and self-variation each. Thus, summation of correct identified items led to a total score of irony detection with a maximum of 40. Subscales were constructed by summation of correct identified items within every item condition ironic praise (IP), ironic criticism (IC), literal criticism (LC), literal praise (LP) with a total maximum of 20 above, and 10 within each context condition. In the main study, all four sets of items were pseudo-randomly combined with all videos, which led to 16 combinations. With a crossover design for stereotype and self-involvement, every participant was pseudo-randomly assigned to two combinations. Thus, every participant participated in two runs. The sequence was counterbalanced among the study participants.

#### Test Construction

The Tuebingen Test of Irony Comprehension was constructed *de novo* for the study. In a pre-study, 110 conversations containing ironic praise (IP), ironic criticism (IC), literal praise (LP), and literal criticism (LC) were presented. Stimuli consist of short statements. They were matched for syntactic structure (one sentence each, ending with a full stop), as well as length. Irony is defined as linguistic irony in this study: a figure of speech, that conveys a different meaning than what has literally been said. Due to often reported “asymmetry of affect” in irony, with ironic praise being less common (68–70) and probably more complex to interpret (70, 71), more items on ironical praise were constructed. Forty-two additional healthy subjects, which did not participate in the main study (mean age = 28.71, *SD* = 7.22, Range = 16–63), assigned all items regarding literality (ironic/literal) as well as intention (praising/critical) on a dichotomous scale and stated their certainty on the decisions on a four-point scale. Only those items that proved to be clearly ironic, literal, critical and praising and obtained highest certainty scores were then selected for further use. This selection was chosen for two reasons. First, it served as validation of our pragmatic definition for item construction. Second, since irony is characterized by a highly subjective nature, only those items were selected, that based on common ground. The final stimulus pool consisted of 80 scenarios and was randomly stratified into four test versions à 20 stimuli, containing five stimuli per condition (ironic praise, ironic criticism, literal criticism, literal praise) each. These versions did not differ in length and grammatic complexity.

With the healthy sample of the main study (*N* = 96, for a detailed description see Table 1), properties of the newly developed tureony were then examined (item statistics for each individual item are presented in Supplementary Table 1). First, Kuder-Richardson Formula 20 (72) was used for estimating internal consistency of the dichotomously rated items. Reliability of all scales was high (KR-20 IC = 0.95; IP = 0.96; LC = 0.98; LP = 0.96) and no inter-item-correlations were <0.30 or negative correlated. In the next step, theoretically postulated discriminant subscales (IC, IP, LC, LP) were tested empirically. For that reason, a confirmatory factor analysis was conducted via Mplus 6. In Mplus, Muthén and Muthén (73) propose the mean and variance adjusted weighted least squares (WLSMV) approach for categorical observed variables. As fit index, WLSMV estimates Weighted Root Mean Square Residual (WRMR) with <0.9, indicating a good model fit (74). In our analysis, the fit for two different models were tested: a general model with one factor defined by all items and a four-factor model with the subscales ironic criticism, ironic praise, literal criticism, literal praise items loading on one factor each. In a preliminary analysis, significant correlation within ironic (IP, IC), and literal (LP, LC) scales, but not within critical (IC, LC) and praising scales (IP, LP) had been shown and were therefore defined in the four-factor model as well. Both models had acceptable fit. The four-factor model (χ^2^ = 1701.28, df = 1704; CFI = 1.0; TLI = 1.01; RMSEA = 0.00, Probability < 0.05 = 1.00; WRMR = 0.83) had slightly better fit indices as the one-factor model (χ^2^ = 1709.06, df = 1710; CFI = 1.0; TLI = 1.00; RMSEA = 0.00, Probability < 0.05 = 0.99; WRMR = 0.84). Even though WLSMV has been reported to be robust for small samples (75), the current sample was explicitly small for a confirmatory factor analysis (below *N* = 200). Thus, results should be regarded cautiously and replicated in a larger sample.

After that, validity of constructed items was elucidated more thoroughly. To examine if ironic items were correctly identified and contained irony, results of the five-point Likert scale for perceived intention were compared between test subscales. Using the average means of the critical (*M* = 1.97, *SD* = 0.38) and praising (*M* = 3.29, *SD* = 0.22) items, a significant difference was found [*t*_(95)_ = −47.05, *p* < 0.001] in paired-sample *t*-test, with each mean being beyond the mid-point of the likert scale ranging from 1 = “critical” to 5 = “praising.” This indicated that every correct identified ironic or literal items was also perceived in the according intention. Then, scoring of perceived intention was used to replicate previously reported property of ironic utterances to be “tinged” with the literal phrase with items in tuerony, showing that ironic utterances in tuerony clearly differed from literal ones and contained irony. For that reason, again, paired-samples *t*-test was conducted on perceived intention of all correct identified items between LC and IC, as well as between LP and IC. Results confirmed that ironic criticism was perceived significantly less critical than literal criticism [*t*_(95)_ = 2.27, *p* < 0.001] and ironic praise was perceived significantly less praising than literal praise [*t*_(95)_ = −12.94, *p* < 0.001].

#### General Data

General intelligence was measured with the German Mehrfachwahl-Wortschatztest (MWT-B), a multiple-choice vocabulary test (76). The MWT-B was applied as paper and pencil test along the collection of demographic data regarding age, gender and education. For a more detailed description of the sample, also the German Versions of the Test of Self-Conscious Affect [TOSCA; (77)] and the State-Trait-Heiterkeits-Inventory [STHI-T <30>; (78)] were applied. Means and standard deviations for all scales and subscales are shown in Table 1.

#### Personality Traits

Metric characteristics of schizotypal personality traits were assessed with the German version of the Schizotypal Personality Questionnaire [SPQ; (79)] by Klein et al. (80). The scale is based on DSM-III-R criteria and contains 74 symptom-related items, with dichotomous yes/no answers. The degree of borderline personality traits were measured with the short version of Borderline Symptom List (BSL-23), based on DSM-IV criteria for Borderline Personality Disorder (81). BSL-23 contains 23 items with self-related statements and is used in clinical and non-clinical samples. Answers were to be given on a five-point Likert scale (ranging from “not at all” = 0 to “a lot” = 4). Autistic personality traits were assessed with the German version (82) of Autism Spectrum Quotient (AQ) by Baron-Cohen et al. (83). The scale is constructed as a self-rating instrument with 50 items on different performance aspects, related to autistic traits. Each item is evaluated on “definitely agree”/”slightly agree” or “slightly disagree”/”definitely disagree.”

#### Mentalization

Measuring affective aspects of mentalization, the Interpersonal Reactivity Index [IRI; (84)] was used as a German short version (85). The IRI consists of 16 items on four subscales: perspective taking (PT), fantasy (FS), empathic concern (EC), and personal distress (PD). For German samples, a general factor for empathy is compounded by a total score of FS, EC, and PD (86). On a five-point Likert scale (ranging from “never” to “always”) participants rate personal statements on perception of empathy. In order to extend the affective components of mentalization on perception of emotions, the Subjective Experience of Emotions Scale (SEE) was deployed. The German scale from Behr and Becker (87) consists of 42 items on perception and evaluation of personal emotions and requires agreement or disagreement on a five-point Likert scale (“not at all” – “true”). Factor analysis in a German sample provided seven subscales: congruence, overwhelming emotions, lack of emotions, symbolization of emotion by bodily experience, symbolization by imagination, regulation of emotions, self-control.

#### Procedure

Permission for the study was obtained from the ethics Committee at the University Clinic of Tübingen. After receiving complete information about the study, subjects gave their written informed consent. Subjects received a 5 Euro compensation for participation.

### Statistical Analysis

Compiled data from the online survey and offline tests were transferred into and processed with IBM SPSS Statistics 24®. Significance level for all statistical calculations was set to *p* < 0.05. First, general performance on tuerony and other demographics of the sample were described. In line with this, *t*-tests were conducted to evaluate the difference between ironic and literal remarks.

In the next step, the role of the context factors “stereotype” and “self-involvement” in irony comprehension were investigated. A two-way multivariate analysis of variance (MANOVA) was conducted, with speaker's stereotype (physician/actor) and participant's self-involvement (neutral observer/direct interaction) as one factor each, personality scores of schizotypy (SPQ-G total score), borderline (BSL-23) and autism (AQ) as covariates, and the general performance on tuerony (total score) as dependent variable.

After that, possible influences of the participants' personality on irony comprehension were analyzed. All personality traits were considered as dimensional predictors in analyses. Due to inter-correlations (BSL-23 with SPQ *r* = 0.65, BSL-23 with AQ *r* = 0.35, SPQ and AQ *r* = 0.57, all *p* < 0.001) separate analyses were run for every personality trait. As general intelligence (MWT-B), gender and age did not correlate with performance on tuerony they were not included as additional covariates.

First, single regressions were conducted to elucidate if there was a general impact of each personality trait (schizotypal, borderline, autistic) on irony detection (total score). All models were calculated using a bootstrap procedure with a sample size *N* = 1,000 (88). If personality traits predicted significant general performance on tuerony in regression analyses, they were subsequently investigated regarding their impact on specific subscales. Thus, a separate multivariate analysis of covariance (MANCOVA) was conducted including respective personality scales as predicting covariate and four subscales of tuerony (IC, IP, LC, LP) as dependent variable each.

Afterwards, the influence of mentalization abilities contributing to performance on irony detection within each relevant personality trait were analyzed. Via median split, the sample was therefore divided into high and low scoring groups for each personality trait separately. Relevant subscales of mentalization were then compared between these groups with *t*-tests. Next, differences between high- and low-scoring subjects within the mentalization scales of SEE and IRI were examined to detect possible scales that might be related to decreased irony comprehension in high scoring individuals. Then, identified mentalization subscales were correlated with the total score of tuerony within the high scoring groups. To elucidate an influence of the respective mentalization scales beyond personality traits, they were finally entered with forced entry in one step in multiple linear regressions predicting the total score of tuerony each.

## Results

### General Performance

The average total score for irony detection in the sample was 36.29 (±2.96) with no significant difference for gender [*t*_(94)_ = 0.37, *p* = 0.71] and no significant correlation with intelligence (*r* = 0.17, *p* = 0.10) and age (*r* = 0.07, *p* = 0.51). Instead, there were significant correlations within ironic (IP, IC, *r* = 0.36, *p* < 0.001) and literal (LP, LC, *r* = 0.40, *p* < 0.001) scales, but not within critical (IC, LC, *r* = 0.09, *p* = 0.372) and praising scales (IP, LP, *r* = −0.03, *p* = 0.777). Thus, in the next step, paired *t*-tests to examine differences in performance between ironic and literal subscales were conducted, comparing IC and LC, as well as IP an LP. Results showed that performance on literal scales were significantly lower than on ironic scales [IC vs. LC *t*_(95)_ = 9.7, *p* < 0.001; IP vs. LP *t*_(95)_ = 3.32, *p* < 0.001], both highlighting the relevance to distinguish literal and ironic remarks in a paradigm of pragmatic cognition. In Supplementary Table 2 results for alternative subscales are provided.

### Self-Involvement, Stereotype, and Irony Detection Accuracy

In two-way MANOVA, using Pillai‘s trace, neither stereotype [*V* = 0.00, *F*_(3, 186)_ = 0.22, *p* = 0.88] nor self-involvement [*V* = 0.01, *F*_(3, 186)_ = 0.66, *p* = 0.58] nor the interaction of stereotype and perspective [*V* = 0.01, *F*_(3, 186)_ = 0.30, *p* = 0.82] had a significant effect on the total score of tuerony, considering borderline, autistic and schizotypal traits as covariates. Mean values and standard deviations are shown in Supplementary Table 3.

### Personality Traits and Irony Detection Accuracy

**Figure 3:**
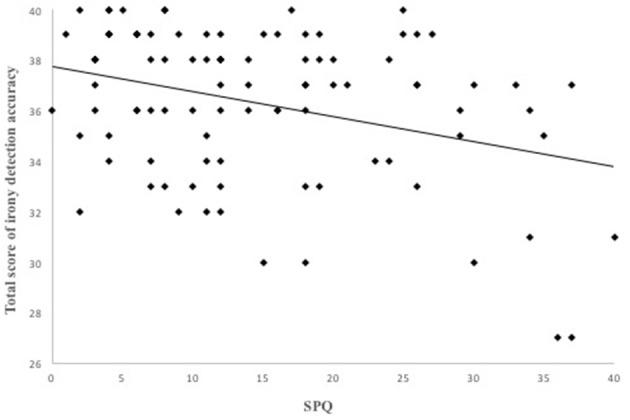
Schizotypal personality traits negatively predicted the total score of irony detection accuracy in *tuerony* in a linear regression.

**Figure 4:**
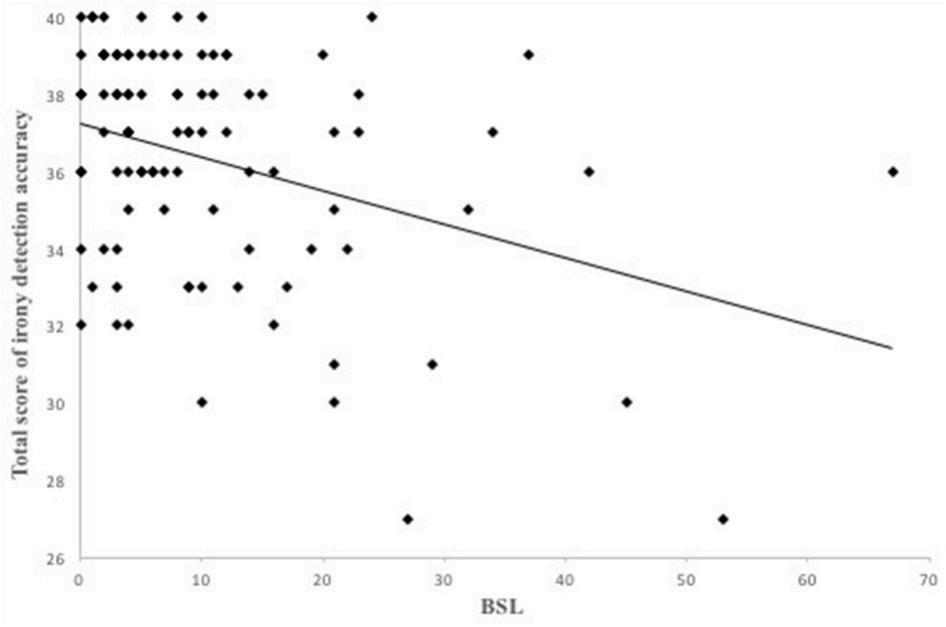
Borderline personality traits negatively predicted the total score of irony detection accuracy in *tuerony* in a linear regression.

Results of the linear regression analyses with schizotypal, borderline and autistic traits are presented in Table 2. Schizotypal and borderline traits both predicted the total score of tuerony (SPQ β = −0.34, *p* < 0.001; BSL-23 β = −0.36, *p* < 0.001) significantly negative, indicating that high expression of schizotypal and borderline traits were associated with lower performance on the detection of ironic and critical remarks (Figures 3, 4). Autistic traits did not contribute significantly to general performance on tuerony (β = −0.09, *p* = 0.104).

**Table 2:** Linear regression analysis of predicted general performance on tuerony by characteristics of schizotypal (SPQ), borderline (BSL-23), and autistic (AQ) personality traits.

	**SPQ**			**BSL-23**			**AQ**		
	**B**	**SE B**	**β**	**B**	**SE B**	**β**	**B**	**SE B**	**β**
Total Score	−0.10	0.03	−0.34^**^	−0.09	0.02	−0.36^***^	−0.09	0.05	−0.17

*** p < 0.001,

** p < 0.01;

*all Durbin-Watson coefficients between d = 1.85 and d = 2.47*.

As regression analyses revealed no significant effect for AQ, only borderline and schizotypal personality traits were included in subsequent MANCOVAs. Results are displayed in Table 3 and revealed a significant negative mutlivariate influence of SPQ on performance on tuerony subscales [*F*_(4, 91)_ = 5.45, *p* = 0.001, Pilai's trace = 0.193, partial η^2^ = 0.19], confirming the previous linear regression in a statistically more valid analysis. Of the separate subscales, only IP [*F*_(1, 94)_ = 12.02, *p* = 0.001, partial η^2^ = 0.113] and LP [*F*_(1, 94)_ = 7.27, *p* = 0.008, partial η^2^ = 0.072] were significantly negative associated with schizotypal personality traits, indicating that individuals with high schizotypy show particular impairments in the detection of praising stimuli, regardless if they are uttered ironically or literally. For borderline symptoms, the results showed a significant negative multivariate influence of the borderline traits on performance on all subscales [*F*_(4, 91)_ = 10.52, *p* < 0.001, Piliai's trace = 0.203, partial η^2^ = 0.316]. Just as in schizotypal traits, BSL-23 was significantly negative associated with IP [*F*_(1, 94)_ = 36.68, *p* < 0.001, partial η^2^ = 0.281]. However, borderline personality traits were neither significantly associated with LP [*F*_(1, 94)_ = 1.97, *p* = 0.164, partial η^2^ = 0.02], nor with LC [*F*_(1, 94)_ = 3.33, *p* = 0.071, partial η^2^ = 0.034]. Instead, borderline personality traits were significantly negative associated with IC [*F*_(1, 94)_ = 5.28, *p* < 0.05, partial η^2^ = 0.053], indicating that healthy adults with high borderline traits might have specific problems in detecting ironic remarks, regardless of their critical or praising intention.

**Table 3:** Results of the two MANCOVAs to predict the influence of borderline (BSL-23) and schizotypal (SPQ) personality traits on tuerony subscales.

	**BSL-23**			**SPQ**		
**Scale**	***F***	***df***	**Partial η^**2**^**	***F***	**df**	**Partial η^**2**^**
IC	5.28^*^	94	0.05	2.9	94	0.03
IP	13.19^***^	94	0.281	12.02^**^	94	0.113
LC	1.97	94	0.02	2.94	94	0.03
LP	3.34	94	0.03	7.27^**^	94	0.072

Personality traits were included as covariates in each in order to measure them dimensionally. IC, ironic criticism; IP, ironic praise; LC, literal criticism; LP, literal praise.

*** p < 0.001,

** p < 0.01,

* *p < 0.05*.

### Mentalization and Irony Detection Accuracy

The median split conducted for schizotypal traits (median SPQ = 12), resulted in groups of 52 low-schizotypal and 44 high-schizotypal individuals. Both groups differed significantly in the total SPQ value [*t*_(60)_ = −13.46, *p* = 0.00]. No significant difference for males and females in the SPQ value was found [*t*_(94)_ = 0.13, *p* = 0.90] and no significant correlation for age (*r* = −0.11, *p* = 0.29) and general intelligence (*r* = −0.20, *p* = 0.05). As depicted in Table 4, *t*-tests revealed five subscales of mentalization with significant differences for high and low schizotypal individuals: personal distress (IRI), congruence (SEE), overwhelming emotions (SEE), lack of emotions (SEE) and symbolization by imagination (SEE). Only personal distress was significantly negative related to general performance on *tuereony* (*r* = −0.33, 95% BCa CI [−0.523, −0.126], *p* = 0.03). The multiple regression analysis had a significant effect [*F*_(2, 93)_ = 6.55, *p* = 0.00] for the proportion of explained variance (*R*^2^ = 0.12). The details are displayed in Table 5. However, only SPQ was significantly predicting the dependent variable (β = −0.28, *t* = −2.55, *p* = 0.01), indicating that specific aspects of mentalization did not explain more than schizotypal personality.

**Table 4:** Differences in mentalization scales (IRI, SEE) between individuals with high and low schizotypal (SPQ) and borderline (BSL-23) traits.

		**Schizotypy**			**Borderline**		
		**Low**	**High**		**Low**	**High**	
		**M(SD)**	**M(SD)**	***t***	**M(SD)**	**M(SD)**	***t***
IRI	Total score	44.19 (5.60)	44.20 (5.36)	−0.01	43.85 (5.60)	44.63 (5.32)	−1.40
	Fantasy	13.46 (3.04)	14.20 (2.98)	−1.21	13.42 (3.09)	14.28 (2.89)	−1.06
	Empathic concern	14.88 (2.33)	14.80 (2.33)	0.19	14.74 (2.35)	14.98 (2.25)	−0.51
	Perspective taking	15.85 (2.39)	15.20 (2.92)	1.18	15.70 (2.66)	15.37 (2.66)	0.6
	Personal distress	9.96 (2.28)	12.25 (3.24)	−3.94^***^	10.11 (2.41)	12.12 (3.25)	−3.47^**^
SEE	Congruence	23.73 (3.35)	21.36 (5.07)	2.65^**^	23.81 (3.40)	21.21 (4.99)	2.91^**^
	Overwhelming emotions	15.42 (4.39)	21.02 (4.39)	−5.28^***^	15.98 (4.83)	20.47 (5.91)	−4.01^***^
	Lack of emotions	10.42 (3.17)	12.41 (4.32)	−2.53^*^	10.51 (3.12)	12.35 (4.40)	−2.3^**^
	Symbolization by bodily experience	22.71 (6.58)	25.02 (5.88)	−1.80	23.36 (7.01)	24.28 (5.44)	−0.72
	Symbolization by imagination	13.65 (5.21)	15.95 (5.53)	−2.10^*^	13.83 (5.59)	15.79 (5.14)	−1.77
	Emotion regulation	12.63 (2.27)	12.05 (3.38)	0.98	12.64 (2.77)	12.02 (2.91)	1.06
	Self-Control	21.17 (3.42)	19.95 (4.17)	1.57	21.09 (3.80)	20.02 (3.78)	1.38

M, Mean; SD, Standard deviation; IRI, Interpersonal Reactivity Index; SEE, Scales for experiencing emotions; SPQ, Schizotypal Personality Questionnaire; BSL-23, Borderline Symptom List 23.

*** p < 0.001,

** p < 0.01,

* *p < 0.05; in case of significant Levene-Tests: the corrected T-value is reported*.

**Table 5:** Relation between schizotypal traits and mentalization.

	**Unstandardized coefficients**		**Standardized coefficients**			
**Predictors**	**B**	**SE_**B**_**	**β**	**R**	***R*^**2**^**	**VIF**
**Step 1**						
Constant	38.75	1.11				
	[36.56, 40.94]					
Total score SPQ	−0.08	0.03	−0.28^*^			1.31
	[−0.149, −0.019]					
Personal distress	−0.11	0.11	−0.11	0.35	0.12	1.31
	[−0.330, 0.108]					

Multiple regression model within high-schizotypal healthy individuals with SPQ and Personal Distress (IRI) as predictors and total score of tuerony as dependent variable, with 95% bias corrected and accelerated confidence intervals based on N = 1,000 bootstrap-sample.

* *p < 0.05; corrected R^2^ = 0.105; Durbin-Watson coefficients d = 2.38*.

Median split of the sample based on the total score of BSL-23 (median = 8) resulted in 53 low-borderline, and 43 high-borderline individuals. There was a significant difference in the BSL total score between both groups [*t*_(51)_ = −8.46, *p* < 0.001]. The characteristics of borderline symptoms were not related with gender [*t*_(94)_ = 0.42, *p* = 0.68], general intelligence (*r* = −0.14, *p* = 0.19), and age (*r* = −0.13, *p* = 0.21). High and low trait borderline individuals showed significant differences in four mentalization subscales: personal distress (IRI), congruence (SEE), overwhelming emotions (SEE), lack of emotions (SEE), all displayed in Table 4. In high borderline individuals, there was no significant correlation between any of those scales and total score on tuerony (all *p* > 0.05). Thus, no additional regression analysis was performed.

## Discussion

The new irony detection test tuerony is intended for use in healthy and clinical populations. The test consists of ironic and literal conversational turns, which can be either critical or praising. Stimuli were developed and matched according to several linguistic criteria and with utmost distinction in terms of both literality (ironic vs. literal) and intention (praise vs. criticism) to ensure that they truly contain verbal irony and praising or critical utterances. A paper and pencil version of the test is provided in Supplementary Test 1 and Supplementary Answer Sheet 1. In the present study, the test was evaluated in a sample of 96 healthy, non-clinical individuals. Moreover, a comprehensive assessment of personality traits and mentalization abilities was applied. The results indicate that in addition to known factors, such as contextual information, personality traits may influence irony detection performance. For the first time, a significant impairment of irony detection was shown in non-clinical adults with high characteristics of borderline personality traits, expanding the known deficits in clinical populations.

### A New Test of Irony Detection Accuracy

The tuerony test comprises four types of items, which vary in literality (ironic vs. literal) and intention (praise vs. criticism). The idea behind this variation is that adding praise and criticism seems to be particularly relevant in the clinical context because problems associated with criticism are part of the clinical picture of numerous mental disorders (89, 90). In our paradigm, the influence of specific syntactic and prosodic hints in ironic utterances was limited by the construction of all items according to the same linguistic criteria. Hence, the performance in irony detection would rely on the systematic variation of intention and literality. Previous irony paradigms that more often rely on ironic criticism or sarcasm are supplemented. By integrating praising and literal remarks, a crossover control condition for the incorrect classification of literal statements as ironic is facilitated. In this regard, sufficient reliability can be shown for all subscales (IC, IP, LC, and LP). These subscales are found to be based on distinct factors, with IC and IP, as well as LC and LP, being intercorrelated. This confirmed the importance of distinguishing between literality (ironic vs. literal) and intention (criticism vs. praise) in the examination of pragmatic and social cognition abilities. Likewise, brain lesion studies have indicated that positive emotional connotation in irony could possibly be more demanding for the medial prefrontal cortex (91). Although the present study is insufficient to assess the role of brain regions, the results provide support for the argument against a positive connotation being generally more difficult.

As expected, the healthy sample showed a high performance because the item construction was based on the common ground of irony perception in healthy adults to enable the detection of possible deficits in clinical populations. However, the scores still showed sufficient variability to indicate interindividual differences in irony detection accuracy. In general, contrary to the expectation, the participants performed better in the detection of irony than in the detection of literal utterances. This finding is in contrast to most previous experimental research on irony comprehension. Nevertheless, it should be noted that lack of significant (92, 93) or even marginally better performance for ironic stimuli (94, 95) has been reported in the literature before. The exact reasons for these different results between studies have yet to be explored. A possible, but from our perspective not very likely, explanation is that the combination with either a critical or a praising intention could make the decision more difficult for literal sentences. Further, theoretically, the explicit instruction to decide on the literality of every item and the dichotomous scaling of this answer could also relate to this. On the one hand, there might be a greater amount of ambiguity, particularly in literal utterances, when the individual is instructed to decide if an item is ironic or not. This is because irony is implemented by the incongruence of the given context sentence (e.g., something good happened to the speaker) and the target sentence (e.g., the conversational partner ironically criticizes the speaker for that). Because it usually violates expectancies, this incongruence may be a “hint” of an ironic utterance when there is an explicit instruction to look for hints. However, such incongruence cannot be found in literal statements, leaving more room for the interpretation of intentions where there might be none. On the other hand, the decision has to be assessed on a dichotomous scale. This scale is a simplification because it ignores the fact that conversational turns can have a “soft” ironic “tongue-in-cheek” and can therefore be both ironic and literal at the same time (14, 17). Thus, it would be interesting to evaluate if continuous scales would influence the irony detection in future studies. Nevertheless, this forced dichotomous response type is chosen for two reasons. First, it simplifies the result score. Second, based on previous experience (51, 96), clinical populations, such as persons with schizophrenia, find it challenging to rate certain degrees of ironic intent, which dramatically increases the test duration.

The second decision for any item was to judge the intention. The test participants rated each item on a five-point scale based on how much they perceived the answer of the speaker as praising or criticizing. In the current study, the results of this rating were used to confirm whether the items, correctly identified on their literality were also classified under the correct intention. This evaluation further considered one of the most common intentions for the use of irony: to politely criticize or praise (97–99). Accordingly, ironic utterances were evaluated as significantly less critical or praising than literal ones. This phenomenon can be seen as an additional validation of the stimuli, as it has been long known in irony research as the “tinge hypothesis” (18, 98, 100, 101) and recently also shown in electrophysiologic reactions (102) claiming that irony mutes the positive or negative information given by the speaker.

### Personality Traits and Irony Detection Accuracy

Apart from evaluating the test in a larger group of healthy subjects, the second main goal of this study was to investigate more thoroughly the factors that may influence irony detection.

In the current study, results confirmed previous studies with schizotypal traits (10, 12) and, for the first time, revealed difficulties in the understanding of irony in healthy adults with borderline traits, showing that the personality of the recipient plays a substantial role in the pragmatic communication process. However, in contrast to our expectations based on studies applying the continuity approach (47) in this sample of healthy adults, autistic traits did not impact the detection of ironic remarks. One explanation might be the complexity of the stimuli and task in the current test. In clinical samples, irony comprehension often been investigated in autism by written stories (34, 103, 104). However, in some studies using a more elaborated design patients with autism did not exhibit more difficulties in irony understanding than healthy controls did (105–107). Above that, choosing a forced choice answering format instead of verbal explanations often facilitates the correct interpretation even in clinically diagnosed samples with autism (106). However, Mathersul et al. (108), applying the video-based as well as forced-choice TASIT on a sample of individuals with high-functioning autism, demonstrated specific deficits in those parts including sarcastic remarks. Nevertheless, all studies did not investigate a subclinical population, resulting in the question whether in the pragmatic domain only a higher degree of autistic traits may lead to impairments in healthy adults. Thus, the often reported reduced performance of irony comprehension in clinical populations raises the question on whether these deficits in irony detection accuracy are categorical or dimensional.

The present study adds to the growing body of literature that assumes a relationship between mentalization abilities and irony detection accuracy. Previous research has repeatedly linked mentalization or ToM abilities to impairments in irony comprehension, particularly in investigating reasons for decreased irony comprehension in clinical populations, with the most research available for autism (1, 30, 106, 109), schizophrenia (59, 110–112), and related personality traits in healthy populations (10–12, 113). Bruntsch et al. (13) emphasized a high interindividual variance in irony comprehension even in healthy populations and suggested several personality traits (schizotypy, histrionic self-presentation, sense of humor, self-esteem, and gelotophobia) as potential explanations for this variance. These findings raise the question as to what degree personality traits or psychopathologic characteristics are distinct from or overlap with mentalizing abilities in irony processing. In the current study, only one subscale of the mentalization assessments of SEE and IRI was associated with irony comprehension in individuals with high schizotypal and borderline personality traits. However, such influence was non-significant compared with the impact of personality traits. This result is in line with the study of Mo et al. (114), who also found that ToM abilities had only a limited influence on irony comprehension in schizophrenic patients. However, these findings are in contrast with several studies that showed a high relevance of mentalization abilities in irony detection in psychiatric patients, such as those with autism (1) and schizophrenia (115). One explanation could be that most of those studies did not directly investigate personality traits and mentalization abilities together, thus impeding a comparison between the two.

### Variation of the Speaker

Communication is always embedded in a context. Hence, this study aimed to investigate how this aspect of context, implemented in the occupation of the speaker, influences irony detection. In the current study in healthy individuals no differences in irony detection was found between individuals with an “ironic occupation” (actors) and those with a “non-ironic occupation” (doctors). This was contrary to most previous studies reporting that the type of occupation essentially influences the interpretation of the possible ironic remark (18, 57–59, 61). One explanation for the difference in results may be the use of only one occupation each and the lack of a non-occupational control condition. Subtended to the other studies, the initial presentation of the occupational stereotype had to be maintained and thus was not changed for a large number of items. Theoretically, this could have led to the fading out of the generated impression from the previous shown video. In contrast, it can be argued that the presentation of the stereotypical character in this study was more elaborate. In other studies, the profession and context story were usually changed for every item, and the test was based only on short written texts (18, 59, 61). In the tuerony, all items are embedded in the same narrative context with one stereotype each, presented in a video. Thus, perhaps not despite but rather because of the more elaborate, ecologically valid narrative of the video sequence and the resulting text messages of different forms of irony, the stereotypical occupation might just not have been so central than in short written text vignettes without much more other information. This would be in line with the results of Bruntsch et al. (13), who, taking an extreme position, considered most contextual information simply as “noise” that distracts rather than serves as an additional resource for interpretation. The present research could not control for this hypothesis because all the stimuli were embedded in one stereotypical context. Thus, future studies could also include video vignettes of other occupations, as well as non-occupational control conditions, to investigate whether stereotypes presented in a more elaborate way, such as through video vignettes, enhance, impair, or do not at all impact the comprehension of ironic remarks. Additionally, the application of even more ecologically valid paradigms in clinical populations might add further insights on the recent limited and rather inconsistent findings on this topic. More respectively, the influence of the occupation of the speaker could theoretically be significant in a patient sample as they have more distinguished experiences in their every day life with one of the stereotypes included in our test, namely physicians. This is currently evaluated in another clinical sample.

### Variation of Self-Involvement

Variation of self-involvement of the addressee of the ironic remark was the second possible influence of context on irony comprehension this study tried to investigate, as it has been suggested by several authors before (62–65). To our knowledge, the present study is the first to compare direct self-involvement with a neutral control condition. However, in this healthy population, no significant differences in detection of irony were found between direct interaction and neutral observation. This may not be the case in clinical populations. For example, in patients with schizophrenia (116) and borderline personality disorder (117), self-involved judgments are attributed aberrantly, e.g., as either hyperbolic self-enhancing or insulting (118, 119). Thus, the obtained result may probably be due to the inclusion of non-clinical individuals. Further research should investigate these differences in patients with schizophrenia, autism, and borderline personality disorder.

### Limitations and Future Directions

We are aware of several limitations within our study. Especially as our test is intended for use in healthy and clinical populations, a larger sample is needed to meet criteria for a full-fledged test construction. Instead, the rationale for sample size in the current study followed the examination of influences on irony detection. Among this group of healthy individuals, performance was not associated with general intelligence, serving as a preliminary result for discriminant validity. The result suggests that it may not be a particular executive form of ability, but the individual's proneness or character constellation affecting the recognition of irony (13). However, in future studies it would be eligible to integrate a more detailed validity analysis, e.g., comparing the current test with other paradigms addressing executive functions and more elaborated intelligence tests than the one addressed in this study, even though it has been shown to be correlate well with premorbid intelligence within clinical samples (76). Further, convergent validity needs to be evaluated, comparing the current with other irony detection paradigms. Not only for the investigation of its clinical relevance, but also for a sufficient variance of performance, the test should be applied and analyzed in clinical populations. Especially in patients with disorder specific experiences ans character constellations, there might be different influences of the test conditions. Applying the paradigm on more extreme groups in irony detection could resolve the problem of the restriction of range found in this healthy sample. As item construction and selection was based on common ground in order to avoid the highly subjective nature of irony detection in the first place, all items in the current sample obtained near-ceiling scores. A sample with higher range could therefore ensure characterizing retest-reliability in future studies. Also, predictive validity would be supported if performance on the test could discriminate between patients and healthy controls. Finally, in this paper we focused only on one side of the communicative channel: the receiver of an ironic statement. However, the active use of irony as a rhetorical figure, might also be influenced by personality traits as recently shown by Bruntsch and Ruch (120).

## Conclusion

Previous research has predominantly focused on two aspects of this issue: the established finding that context influences the ease and accuracy of irony detection, and the relationship between irony detection accuracy and the “mentalization” capabilities of the individual. Whereas, the first aspect influenced the field of linguistics, the latter has dominated the research on the relationship between irony detection accuracy and mental disorders. However, as the current results suggest, bringing into focus only the conversational context or mentalization skills might be too limited. The present work proposes a third aspect that could be of major relevance to irony detection accuracy: the individual personality of the recipient.

## Author Contributions

All authors listed have made a substantial, direct and intellectual contribution to the work, and approved it for publication.

### Conflict of Interest Statement

The authors declare that the research was conducted in the absence of any commercial or financial relationships that could be construed as a potential conflict of interest.

## Supplementary Material

Supplementary Table 1 Item statistics for healthy individuals (*N* = 96) in the irony detection accuracy test tuerony. Percentage of correct answers and item statistics for single items within the categories ironic criticism, ironic praise, literal criticism, and literal praise are presented. Items with 100% of correct answers were excluded from item statistics (ironic criticism: item 2, 4, 5, 7, 9, 11, 17, and 20; ironic praise: item 2, 3, 4, 5, 6, 12, 13, and 18; literal praise: tem 2, 6, and 18).

Supplementary Table 2 Additional analyses for alternative subscales, considering literality and irony as separate values. Mean values and standard deviations for self-involvement (neutral observer/direct interaction) and stereotypes (doctor/actor) on irony detection accuracy for the total population (*N* = 96).

Supplementary Table 3 Performance of healthy individuals in the irony detection accuracy test tuerony. Survey of total scores, subscores, and perceived criticism and praise in the total sample (*N* = 96) and specified for females (*n* = 59) and males (*n* = 37).

Supplementary Answer Sheet 1 A simplified version of the original answer sheet, containing a dichotomous scale (ironic vs. literal) and a five-point smiley-based Likert scale (critical to praising).

Supplementary Test 1 One version of the Tuebingen Test of Irony Detection Accuracy with the conditions actress and neutral observer. The test follows the presentation of Supplementary Video 1.

Supplementary Video 1 Video instruction for the Tuebingen Test of Irony Detection Accuracy presenting the social situation in a variation of perspective (direct/neutral observation) and speaker's occupation (doctor/actor). The video shows a combination of the conditions actress (speaker‘s occupation) and neutral observer (perspective).
