# Supplementary material for: A New Test for Irony Detection: The Influence of Schizotypal, Borderline, and Autistic Personality Traits
Source: Front Psychiatry. 2019 Feb 14;10:28. doi: 10.3389/fpsyt.2019.00028 (PMC6382691; doi:10.3389/fpsyt.2019.00028)
Supplement: Supplementary Table 1 — Item statistics for healthy individuals (N = 96) in the irony detection accuracy test tuerony. Percentage of correct answers and item statistics for single items within the categories ironic criticism, ironic praise, literal criticism, and literal praise are presented. Items with 100% of correct answers were excluded from item statistics (ironic criticism: item 2, 4, 5, 7, 9, 11, 17, and 20; ironic praise: item 2, 3, 4, 5, 6, 12, 13, and 18; literal praise: tem 2, 6, and 18). [file Table_1.pdf]

Supplementary Material

A new test for irony detection: the influence of schizotypal, borderline and autistic personality traits

Carolin Kieckhäfer, Anne Felsenheimer, Alexander Rapp*

*** Correspondence:** Corresponding Author: Alexander.Rapp@med.uni-tuebingen.de

Supplementary Table 1: Item statistics for healthy individuals (N = 96) in the irony detection accuracy test *tuerony*. Percentage of correct answers and item statistics for single items within the categories ironic criticism, ironic praise, literal criticism and literal praise are presented. Items with 100% of correct answers were excluded from item statistics (ironic criticism: item 2, 4, 5, 7, 9, 11, 17 and 20; ironic praise: item 2, 3, 4, 5, 6, 12, 13 and 18; literal praise: tem 2, 6 and 18).

	Item	*P*	MID	VID	ITC	α	N
**IC**							
	**1 abholen**	91.7	11.52	3.53	.848	.946	48
	**3 Besuch**	97.9	11.58	4.04	.764	.949	48
	**6 Frankreich**	95.8	11.56	3.78	.860	.945	48
	**8 Kneipe**	89.6	11.50	3.49	.793	.949	48
	**10 Krawatte**	97.9	11.58	4.04	.764	.949	48
	**12 müde**	91.7	11.52	3.53	.848	.946	48
	**13 Paris**	95.8	11.56	3.78	.860	.945	48
	**14 Pizza**	89.6	11.50	3.49	.793	.949	48
	**15 Problem**	97.9	11.58	4.04	.764	.949	48
	**16 Schlaf**	97.9	11.58	4.04	.764	.949	48
	**18 Stadt**	95.8	11.56	3.78	.860	.945	48
	**19 Wäsche**	97.9	11.58	4.04	.764	.949	48
**IP**							
	**1 Gewicht**	93.8	11.58	4.21	.944	.955	48
	**7 Einkauf**	89.6	11.54	4.13	.790	.962	48
	**8 Filmfest**	93.8	11.58	4.21	.944	.955	48
	**9 Geburtstag**	93.8	11.58	4.21	.944	.955	48
	**10 Goethe**	97.9	11.63	4.75	.702	.963	48
	**11 Gut**	89.6	11.54	4.13	.790	.962	48
	**14 Marathon**	97.9	11.63	4.75	.702	.963	48
	**15 Nummer**	95.8	11.60	4.46	.838	.959	48
	**16 Prüfung**	97.9	11.63	4.75	.702	.963	48
	**17 Quiz**	97.9	11.63	4.75	.702	.963	48
	**19 Screening**	93.8	11.58	4.21	.944	.955	48
	**20 Zimmer**	93.8	11.58	4.21	.944	.955	48
**LC**							
	**1 Ausgesperrt**	89.6	33.50	42.51	.745	.975	48
	**2 Blitzen**	83.3	33.56	41.23	.875	.974	48
	**3 Chef**	54.2	33.85	40.77	.713	.976	48
	**4 Fahrrad**	85.4	33.54	41.57	.849	.974	48
	**5 Freibad**	89.6	33.50	42.51	.745	.975	48

	**6 Haarfarbe**	60.4	33.79	40.38	.793	.975	48
	**7 Hund**	81.3	33.58	40.97	.887	.974	48
	**8 Kaffee**	58.3	33.81	40.45	.774	.975	48
	**9 Kasse**	79.2	33.60	40.76	.894	.973	48
	**10 Koch**	58.3	33.81	40.45	.774	.975	48
	**11 Frisur**	66.7	33.73	40.29	.842	.974	48
	**12 Party**	77.1	33.63	40.58	.895	.973	48
	**13 Pflanze**	85.4	33.54	41.57	.849	.974	48
	**14 Serie**	75.0	33.65	40.45	.893	.973	48
	**15 Sorry**	68.8	33.71	40.30	.856	.974	48
	**16 Steaks**	75.0	33.65	40.45	.893	.973	48
	**17 Stinken**	89.6	33.50	42.51	.745	.975	48
	**18 Arbeit**	93.8	33.46	43.70	.571	.976	48
	**19 Verkäufer**	85.4	33.54	41.57	.849	.974	48
	**20 Vortrag**	83.3	33.56	41.23	.875	.974	48
**LP**							
	**1 Apfel**	97.9	30.46	13.36	.583	.958	48
	**3 Fehler**	85.4	30.58	11.74	.872	.953	48
	**4 Feierabend**	97.9	30.46	13.36	.583	.958	48
	**5 Gehalt**	83.3	30.60	11.65	.858	.954	48
	**7 Leute**	95.8	30.48	12.89	.732	.956	48
	**8 Marathon**	81.3	30.63	11.60	.833	.954	48
	**9 Nichte**	81.3	30.63	11.60	.833	.954	48
	**10 Papier**	79.2	30.65	11.64	.782	.956	48
	**11 Politik**	93.8	30.50	12.55	.798	.955	48
	**12 Route**	95.8	30.48	12.89	.732	.956	48
	**13 Ruhe**	95.8	30.48	12.89	.732	.956	48
	**14 Tag**	91.7	30.52	12.26	.850	.954	48
	**15 Text**	87.5	30.56	11.87	.875	.953	48
	**16 Unterlage**	97.9	30.46	13.36	.583	.958	48
	**17 Wandern**	89.6	30.54	12.04	.868	.953	48
	**19 Zeit**	97.9	30.46	13.36	.583	.958	48
	**20 Zigaretten**	91.7	30.52	12.26	.850	.954	48

Note. P = percentage of correct answers, MID = scale mean if item deleted, VID = scale variance if item deleted, ITC = corrected item total correlation, α = Cronbach`s α if item is deleted, IC = Ironic criticism, IP = Ironic praise, LC = Literal criticism, LP = Literal praise.
