# Supplementary material for: A New Test for Irony Detection: The Influence of Schizotypal, Borderline, and Autistic Personality Traits
Source: Front Psychiatry. 2019 Feb 14;10:28. doi: 10.3389/fpsyt.2019.00028 (PMC6382691; doi:10.3389/fpsyt.2019.00028)
Supplement: Supplementary Table 2 — Additional analyses for alternative subscales, considering literality and irony as separate values. Mean values and standard deviations for self-involvement (neutral observer/direct interaction) and stereotypes (doctor/actor) on irony detection accuracy for the total population (N = 96). [file Table_2.pdf]

**Supplementary Table 2**: Additional analyses for alternative subscales, considering literality and irony as separate values. Mean values and standard deviations for self-involvement (neutral observer/direct interaction) and stereotypes (doctor/actor) on irony detection accuracy for the total population (N = 96).

| | **perspective** | | | | **stereotype** | | | | |
|---|---|---|---|---|---|---|---|---|---|
| | **self-involvement** | | **neutral** | | **doctor** | | **actor** | | **Max. i** |
| **Irony** | M | SD | M | SD | M | SD | M | SD | |
| **Total** | 18.14 | 1.70 | 18.16 | 1.80 | 18.22 | 1.76 | 18.07 | 1.73 | 20 |
| **I** | 9.67 | .72 | 9.71 | .66 | 9.70 | .73 | 9.68 | .66 | 10 |
| **L** | 8.47 | 1.43 | 8.45 | 1.68 | 8.52 | 1.55 | 8.40 | 1.57 | 10 |

*Note.* I = Ironic, L = Literal, Max. i = maximum number of correct items.
