# Supplementary material for: A New Test for Irony Detection: The Influence of Schizotypal, Borderline, and Autistic Personality Traits
Source: Front Psychiatry. 2019 Feb 14;10:28. doi: 10.3389/fpsyt.2019.00028 (PMC6382691; doi:10.3389/fpsyt.2019.00028)
Supplement: Supplementary Table 3 — Performance of healthy individuals in the irony detection accuracy test tuerony. Survey of total scores, subscores, and perceived criticism and praise in the total sample (N = 96) and specified for females (n = 59) and males (n = 37). [file Table_3.pdf]

Supplementary Table 3: Performance of healthy individuals in the irony detection accuracy test *tuerony*. Survey of total scores, subscores and perceived criticism and praise in the total sample (N = 96) and specified for females (n = 59) and males (n = 37).

	**female**		**male**		**total sample**		**Max. score**
**Irony**	M	SD	M	SD	M	SD	and
**Total**	36.20	3.04	36.43	2.84	36.29	2.96	40
**Irony**	19.31	1.25	19.49	.87	19.38	1.12	20
**IC**	9.64	.71	9.78	.53	9.70	.65	10
**IP**	9.66	.78	9.70	.57	9.68	.70	10
**Literal**	16.90	2.62	16.95	2.72	16.92	2.64	20
**LC**	7.68	1.80	7.73	2.26	7.70	1.97	10
**LP**	9.22	1.22	9.22	1.00	9.22	1.14	10
**Critical**	17.32	1.99	17.51	2.38	17.40	2.14	20
**Praising**	18.88	1.44	18.92	1.12	18.90	1.32	20
**LC**	7.68	1.80	7.73	2.26	7.70	1.97	10
**LP**	9.22	1.22	9.22	1.00	9.22	1.14	10
**C Valence**	1.98	.39	1.94	.36	1.97	.38	1
**P Valence**	3.31	.23	3.25	.22	3.29	.22	5

Note. IC = Ironic criticism, IP = Ironic praise, LC = Literal criticism, LP = Literal praise, C Valence = perceived criticism, P Valence = perceived praise; Max. items = maximum of score.
