# Supplementary material for: A New Test for Irony Detection: The Influence of Schizotypal, Borderline, and Autistic Personality Traits
Source: Front Psychiatry. 2019 Feb 14;10:28. doi: 10.3389/fpsyt.2019.00028 (PMC6382691; doi:10.3389/fpsyt.2019.00028)
Supplement: Supplementary Answer Sheet 1 — A simplified version of the original answer sheet, containing a dichotomous scale (ironic vs. literal) and a five-point smiley-based Likert scale (critical to praising). [file Data_Sheet_1.PDF]

Antwortbogen für den *tuerony*

Bitte kreuze an, ob Lauras Antwort auf deine Nachricht
ironisch oder **wörtlich** und zusätzlich ob sie **kritisierend** oder **lobend** war:

			**kritisch**				**lobend**
1.	ironisch	wörtlich	☹	☹	😐	☺	☺
2.	ironisch	wörtlich	☹	☹	😐	☺	☺
3.	ironisch	wörtlich	☹	☹	😐	☺	☺
4.	ironisch	wörtlich	☹	☹	😐	☺	☺
5.	ironisch	wörtlich	☹	☹	😐	☺	☺
6.	ironisch	wörtlich	☹	☹	😐	☺	☺
7.	ironisch	wörtlich	☹	☹	😐	☺	☺
8.	ironisch	wörtlich	☹	☹	😐	☺	☺
9.	ironisch	wörtlich	☹	☹	😐	☺	☺
10.	ironisch	wörtlich	☹	☹	☹	☺	☺
11.	ironisch	wörtlich	☹	☹	😐	☺	☺
12.	ironisch	wörtlich	☹	☹	😐	☺	☺
13.	ironisch	wörtlich	☹	☹	😐	☺	☺
14.	ironisch	wörtlich	☹	☹	😐	☺	☺
15.	ironisch	wörtlich	☹	☹	😐	☺	☺
16.	ironisch	wörtlich	☹	☹	😐	☺	☺
17.	ironisch	wörtlich	☹	☹	😐	☺	☺
18.	ironisch	wörtlich	☹	☹	😐	☺	☺
19.	ironisch	wörtlich	☹	☹	😐	☺	☺
20.	ironisch	wörtlich	☹	☹	😐	☺	☺
