# Supplementary material for: A New Test for Irony Detection: The Influence of Schizotypal, Borderline, and Autistic Personality Traits
Source: Front Psychiatry. 2019 Feb 14;10:28. doi: 10.3389/fpsyt.2019.00028 (PMC6382691; doi:10.3389/fpsyt.2019.00028)
Supplement: Supplementary Test 1 — One version of the Tuebingen Test of Irony Detection Accuracy with the conditions actress and neutral observer. The test follows the presentation of Supplementary Video 1. [file Data_Sheet_2.PDF]

Nach eurem Treffen beginnen Stefan und Laura zu chatten.

Im folgenden siehst du ein Handydisplay, welches zufällige Ausschnitte aus **dem Chatverlauf mit Laura** anzeigt.

Schätze dann ein, ob **Lauras Antwort auf Stefans Nachricht:**

ironisch oder **wörtlich**
kritisierend oder **lobend** war.

Notiere die Antwort jeweils auf deinem Antwortbogen.

1

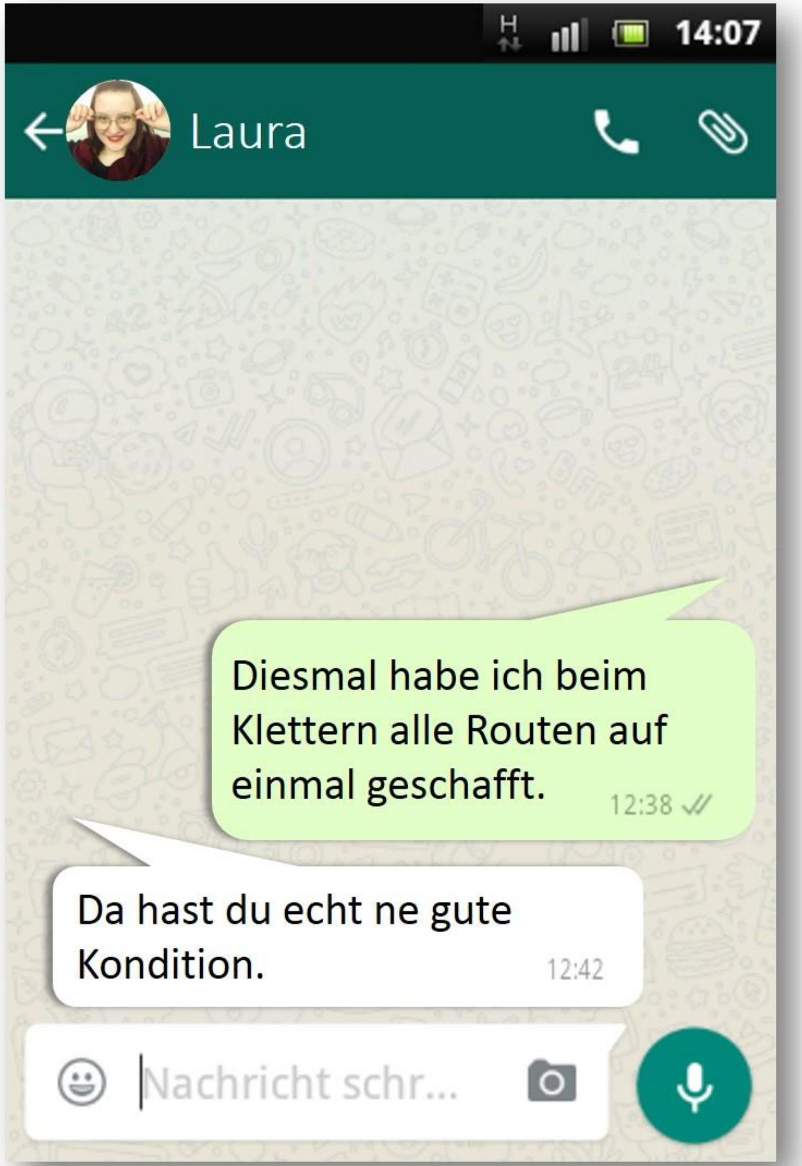
14:07
Laura
Diesmal habe ich beim Klettern alle Routen auf einmal geschafft.
12:38
Da hast du echt ne gute Kondition.
12:42
Nachricht schr...

2

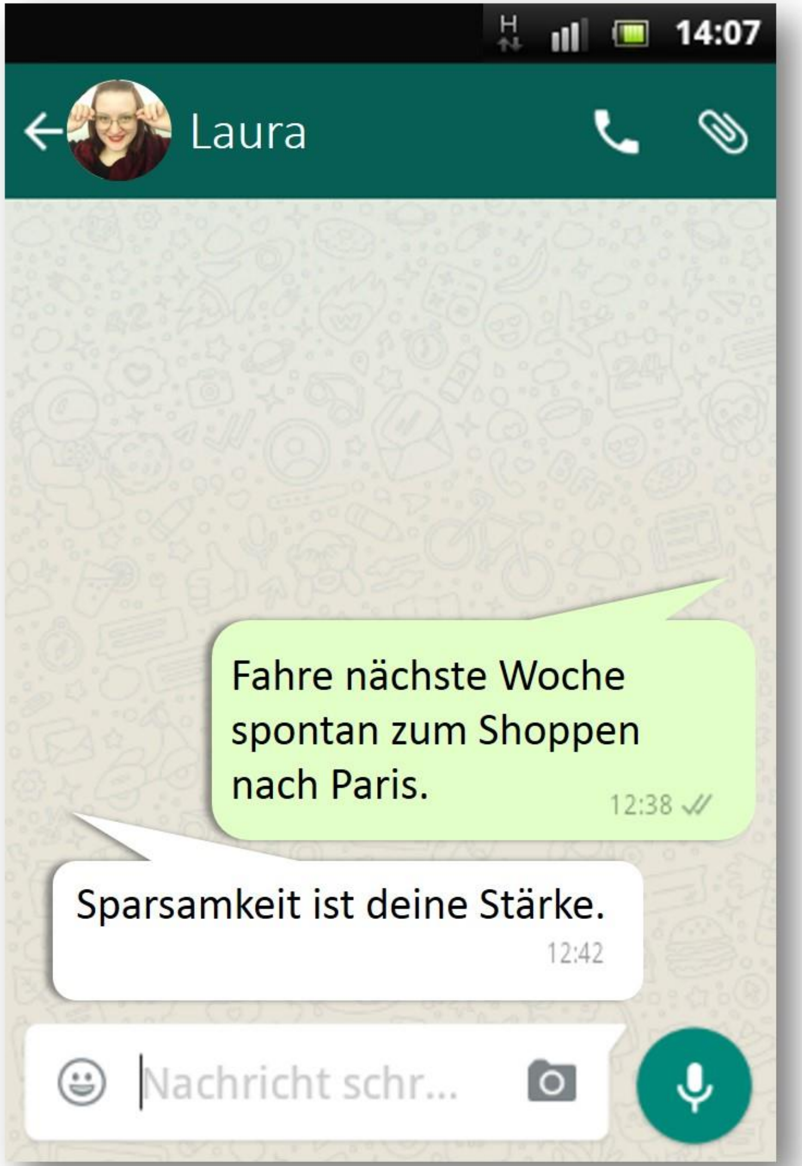
14:07
Laura
Fahre nächste Woche spontan zum Shoppen nach Paris.
12:38
Sparsamkeit ist deine Stärke.
12:42
Nachricht schr...

3

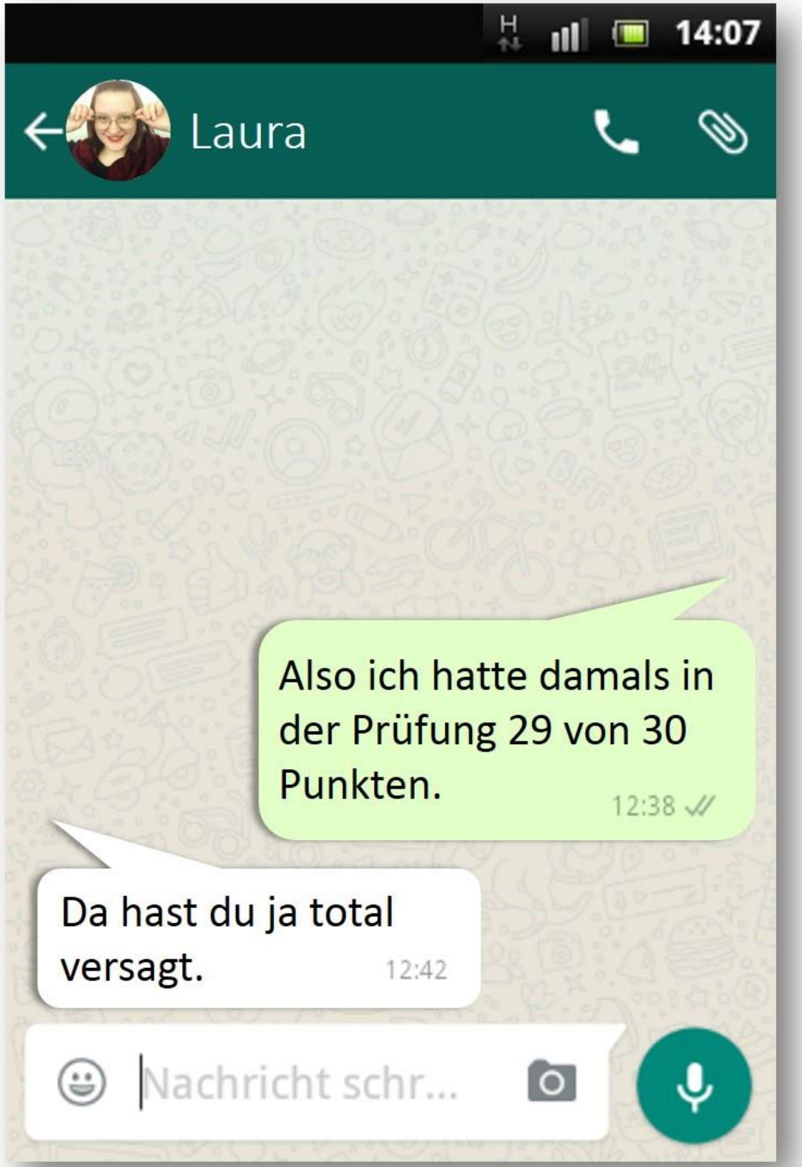
14:07
Laura
Also ich hatte damals in der Prüfung 29 von 30 Punkten.
12:38
Da hast du ja total versagt.
12:42
Nachricht schr...

4

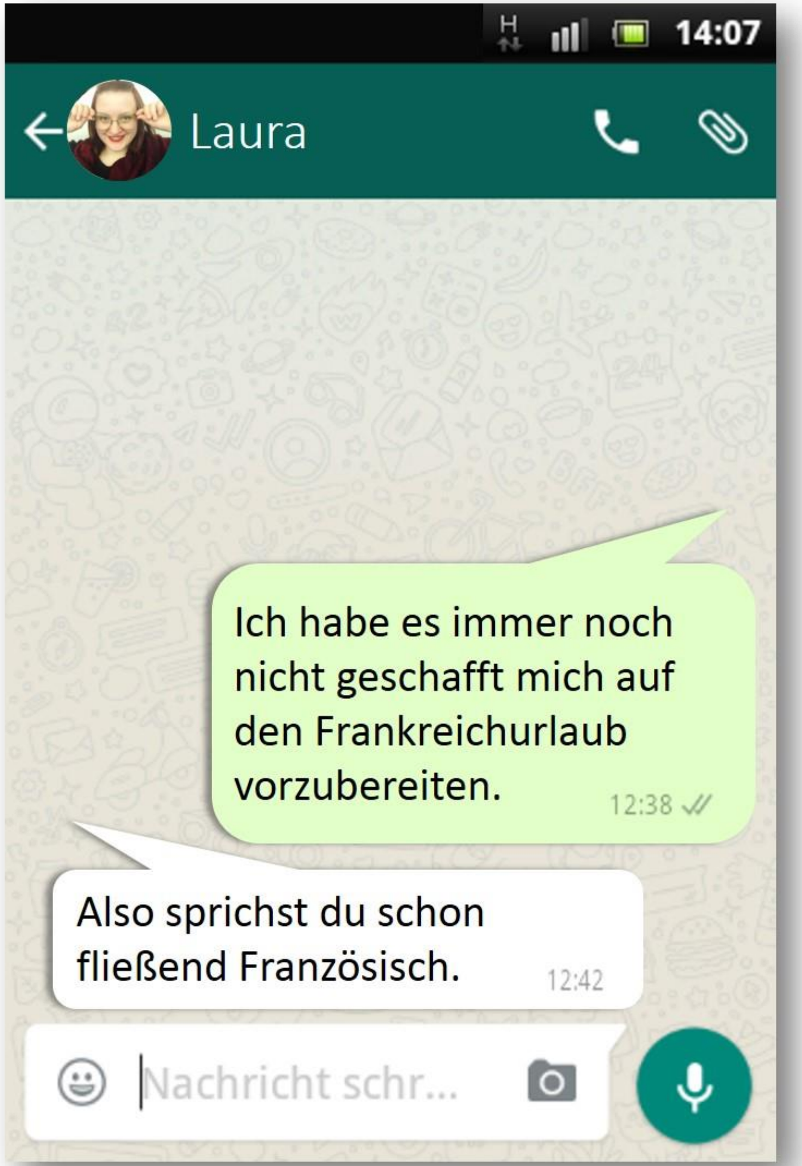
14:07
Laura
Ich habe es immer noch nicht geschafft mich auf den Frankreichurlaub vorzubereiten.
12:38
Also sprichst du schon fließend Französisch.
12:42
Nachricht schr...

5

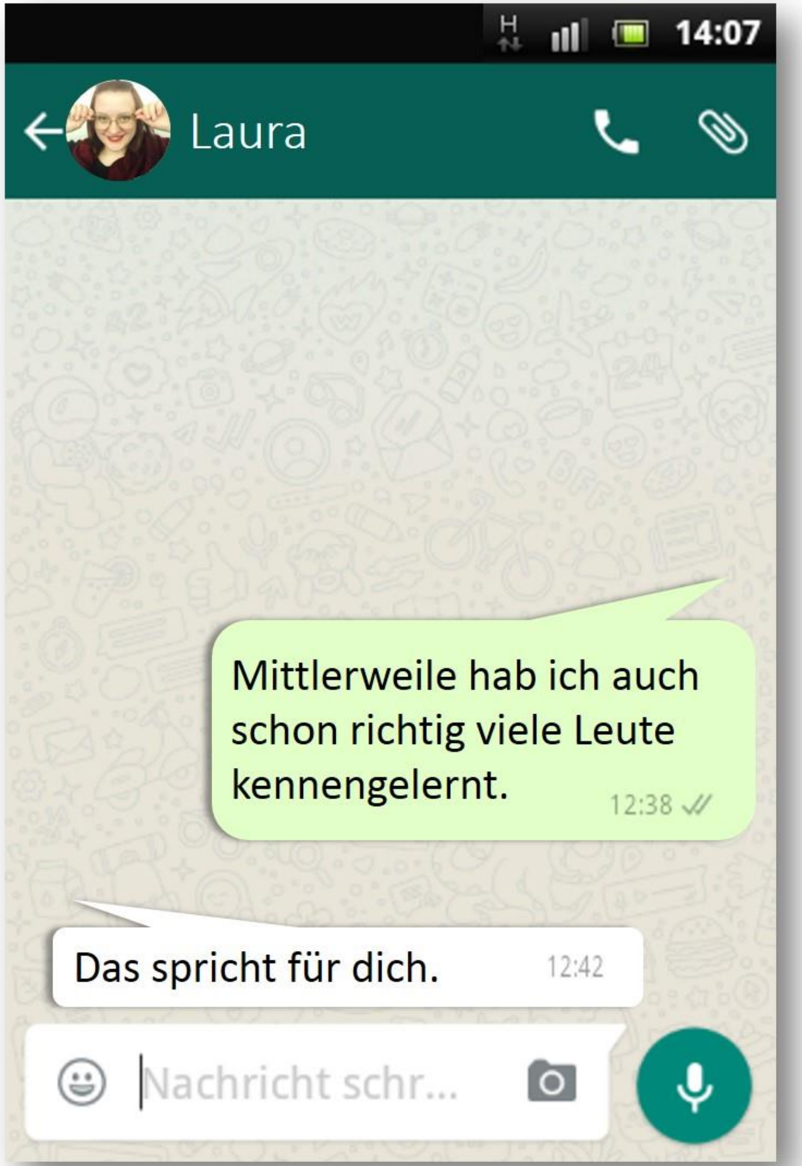
14:07
Laura
Mittlerweile hab ich auch schon richtig viele Leute kennengelernt.
12:38
Das spricht für dich.
12:42
Nachricht schr...

6

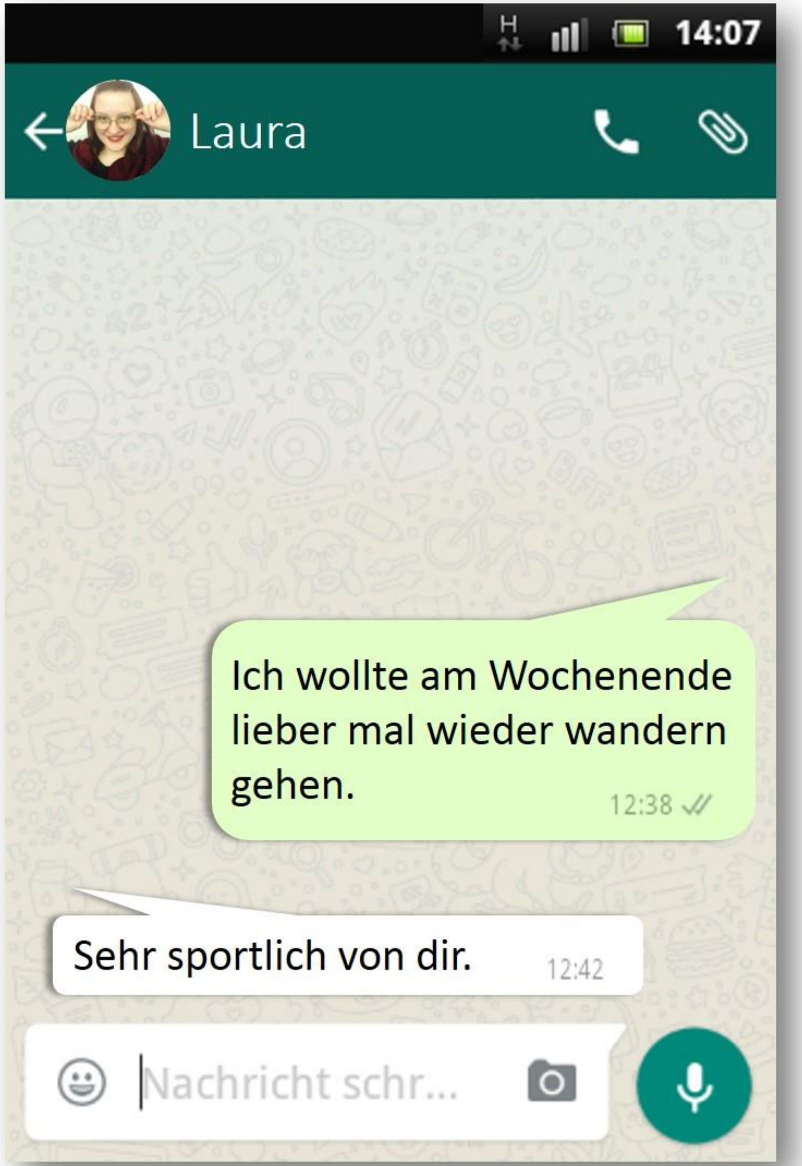
14:07
Laura
Ich wollte am Wochenende lieber mal wieder wandern gehen.
12:38
Sehr sportlich von dir.
12:42
Nachricht schr...

7

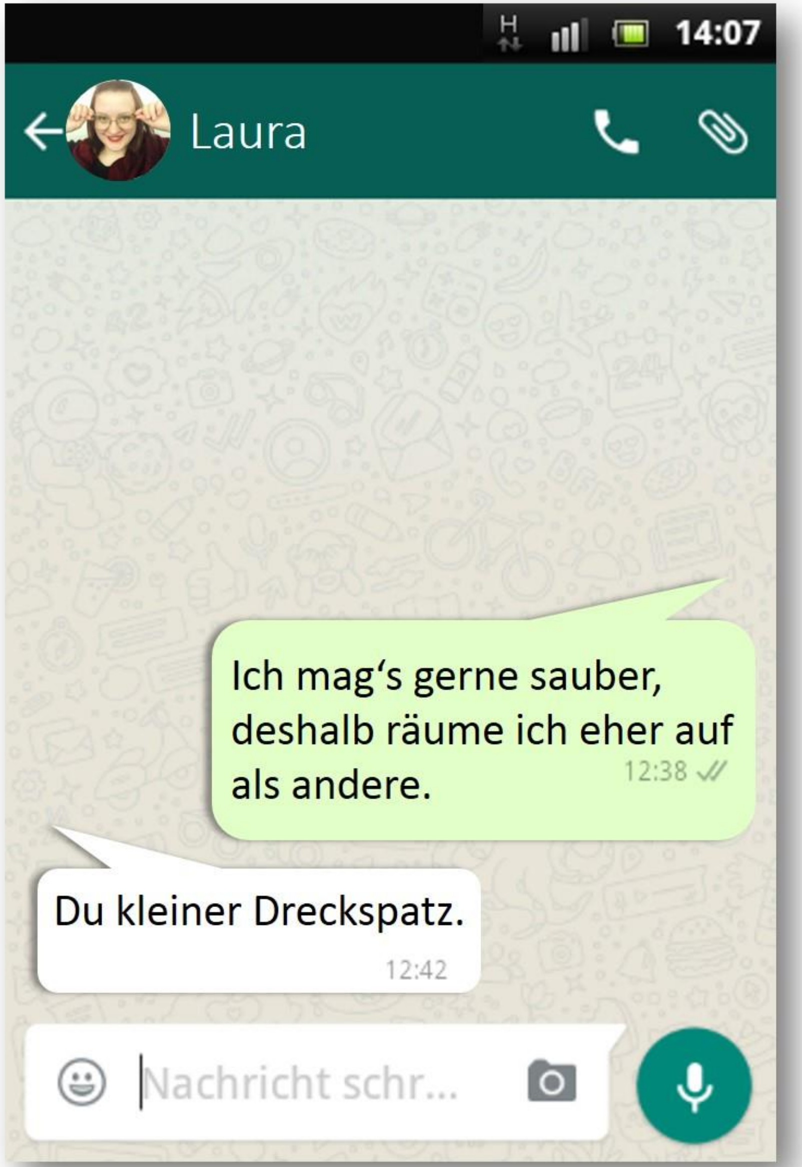
14:07
Laura
Ich mag‘s gerne sauber, deshalb räume ich eher auf als andere.
12:38
Du kleiner Dreckspatz.
12:42
Nachricht schr...

8

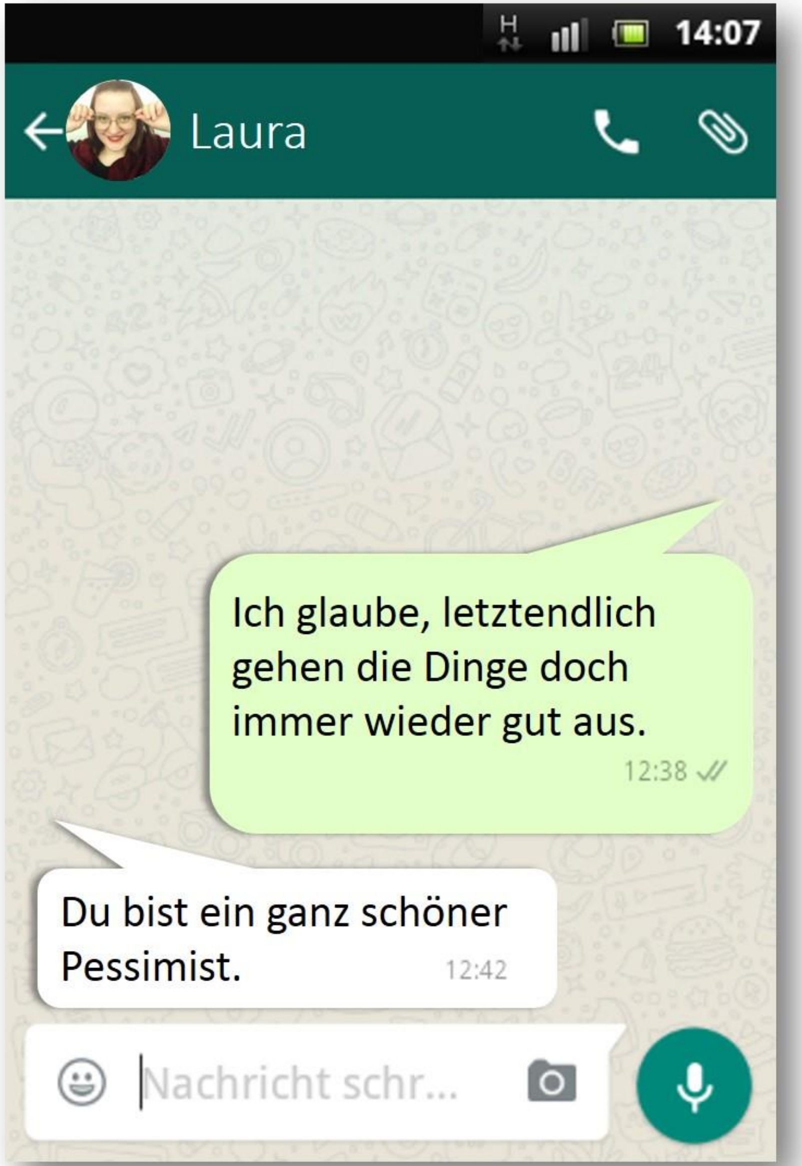
14:07
Laura
Ich glaube, letztendlich gehen die Dinge doch immer wieder gut aus.
12:38
Du bist ein ganz schöner Pessimist.
12:42
Nachricht schr...

9

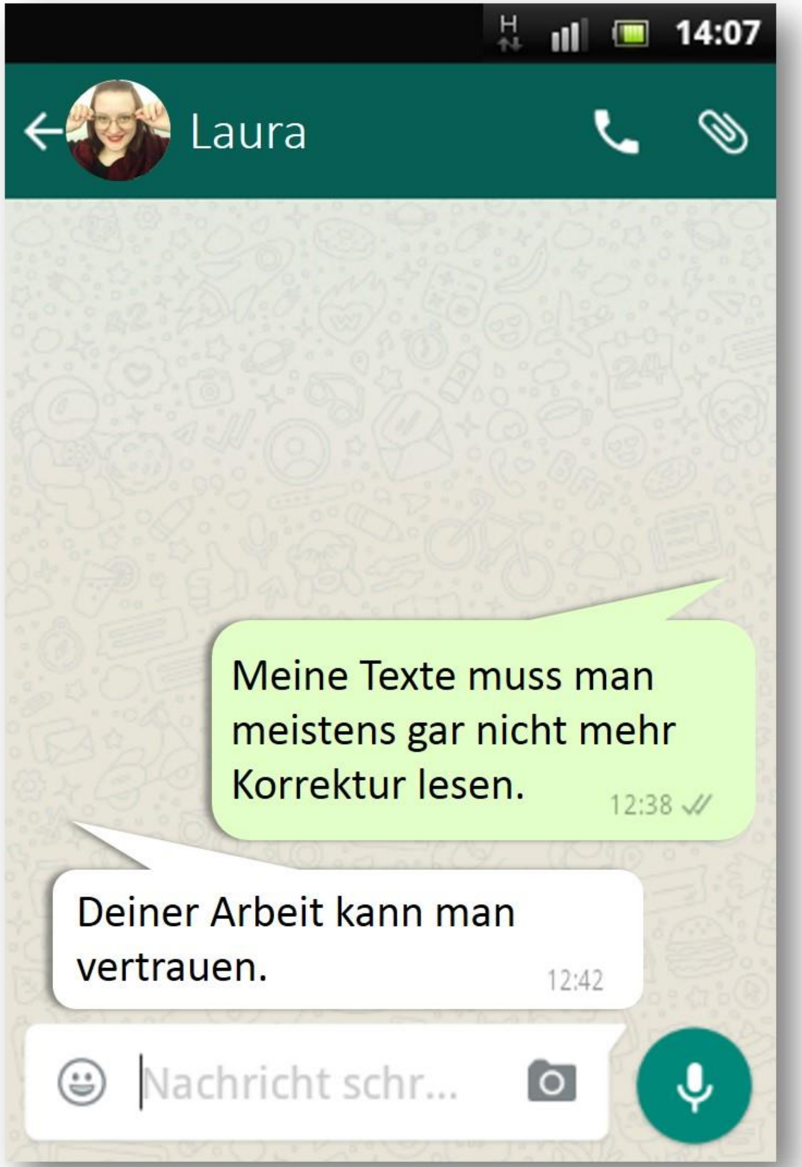
14:07
Laura
Meine Texte muss man meistens gar nicht mehr Korrektur lesen.
12:38
Deiner Arbeit kann man vertrauen.
12:42
Nachricht schr...

10

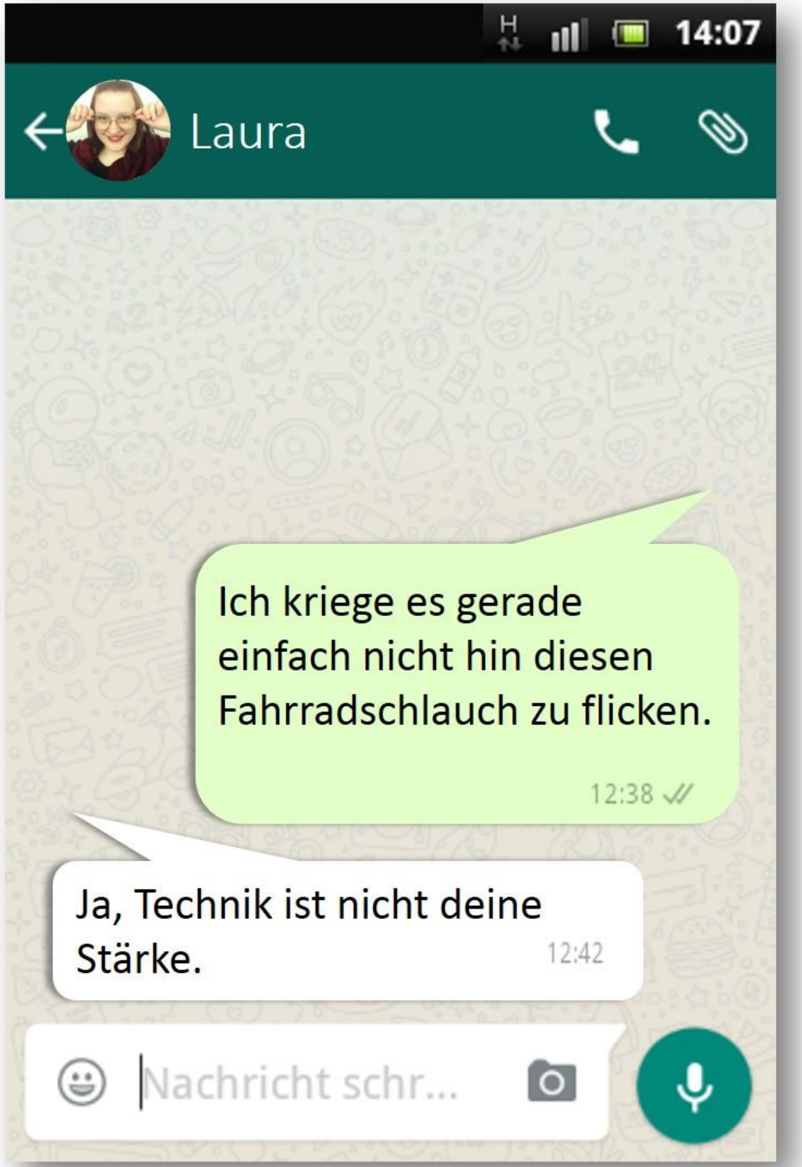
14:07
Laura
Ich kriege es gerade einfach nicht hin diesen Fahrradschlauch zu flicken.
12:38
Ja, Technik ist nicht deine Stärke.
12:42
Nachricht schr...

11

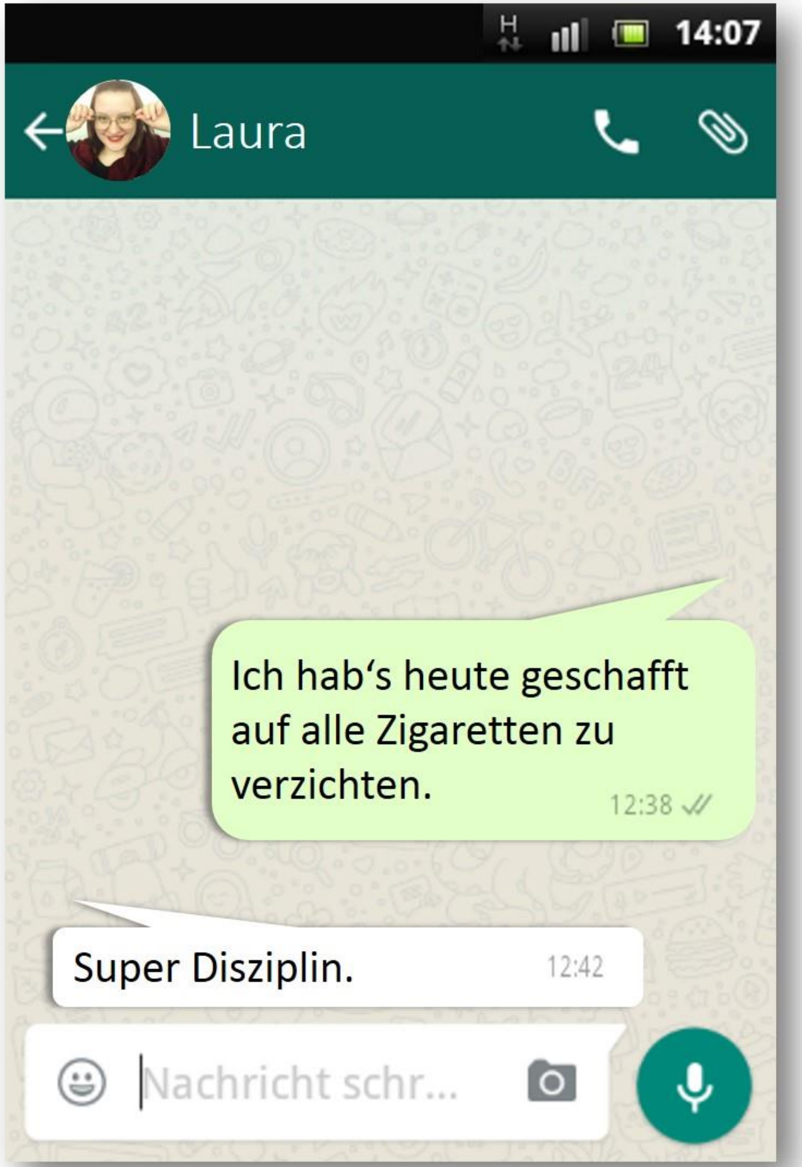
14:07
Laura
Ich hab‘s heute geschafft auf alle Zigaretten zu verzichten.
12:38
Super Disziplin.
12:42
Nachricht schr...

12

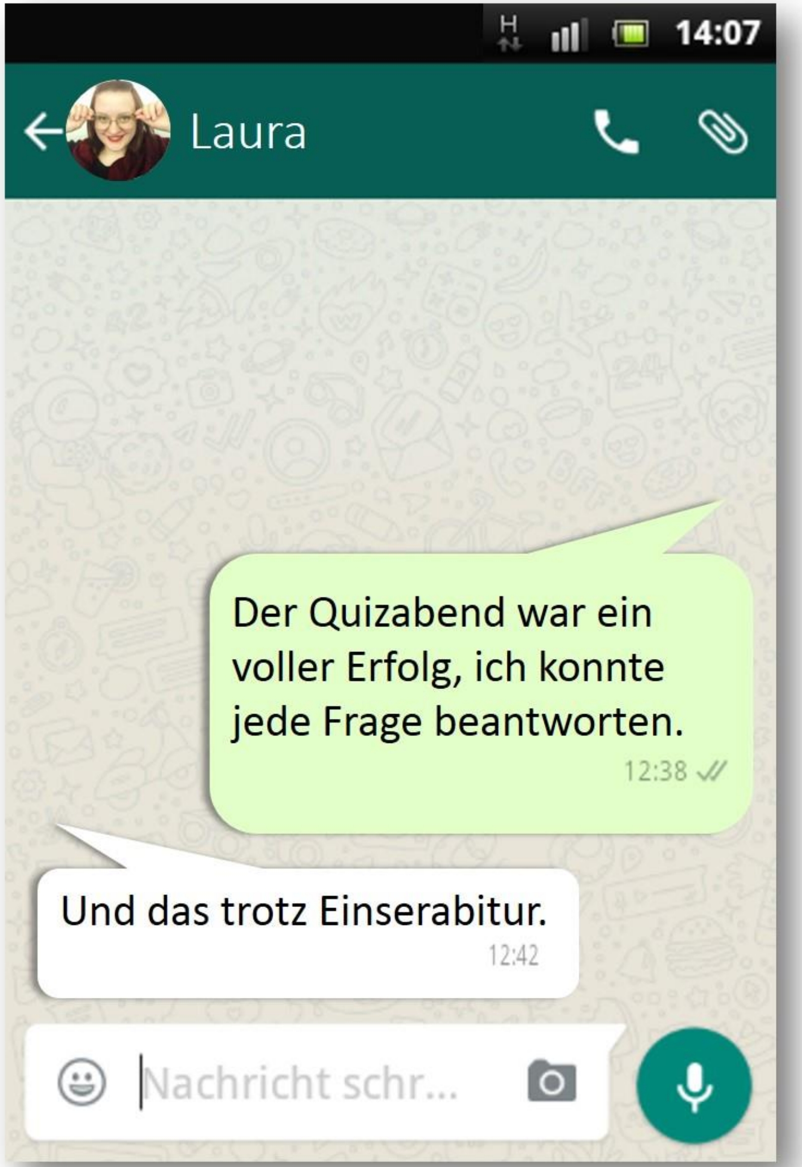
14:07
Laura
Der Quizabend war ein voller Erfolg, ich konnte jede Frage beantworten.
12:38
Und das trotz Einserabitur.
12:42
Nachricht schr...

13

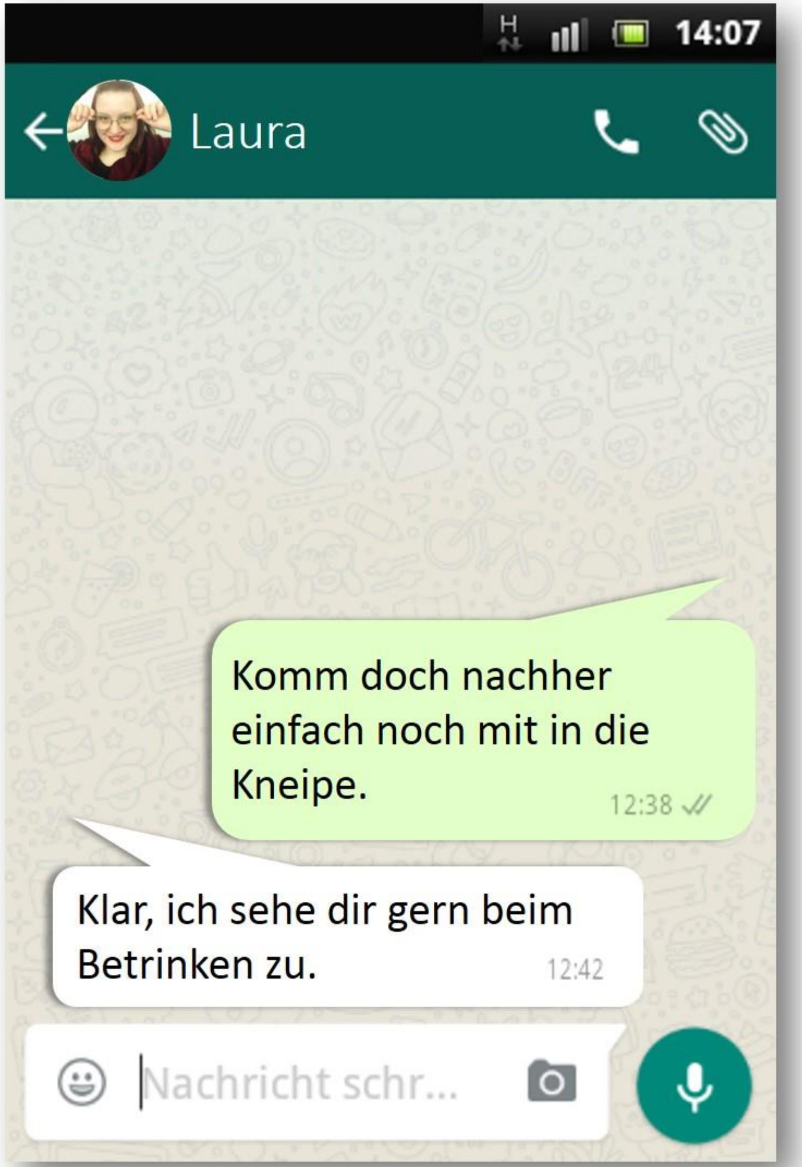
14:07
Laura
Komm doch nachher einfach noch mit in die Kneipe.
12:38
Klar, ich sehe dir gern beim Betrinken zu.
12:42
Nachricht schr...

14

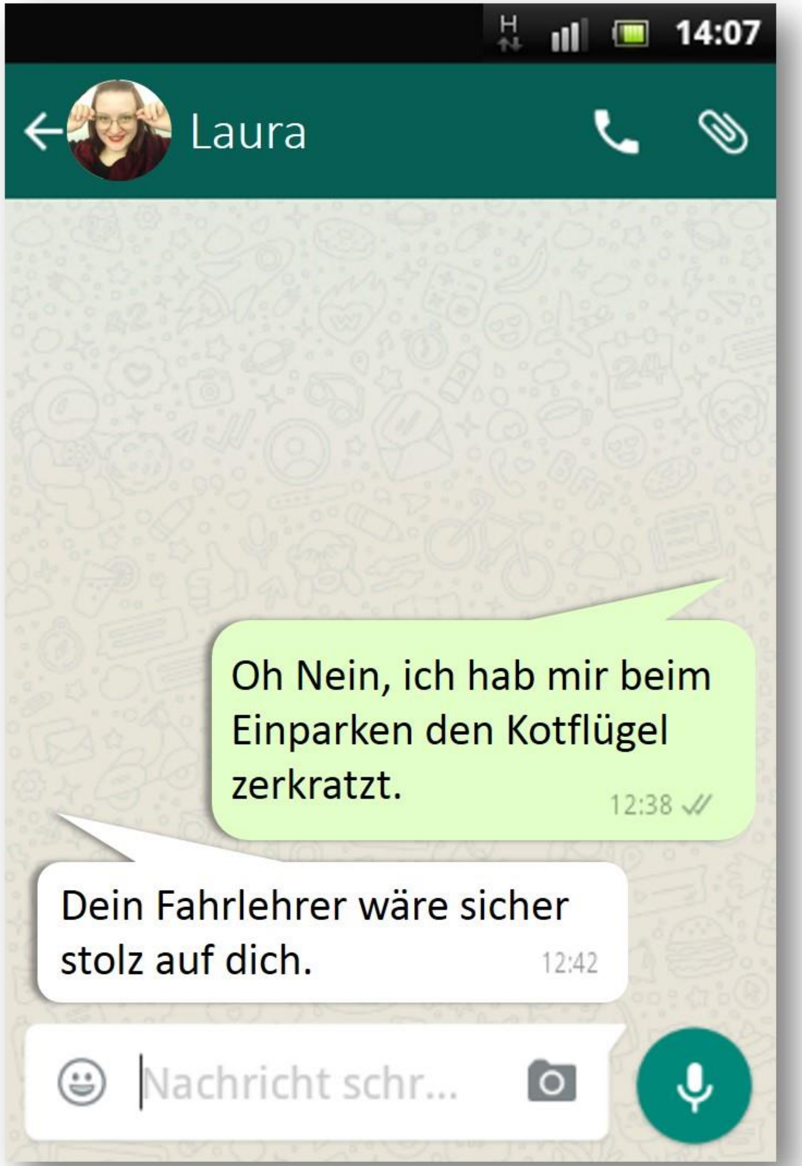
14:07
Laura
Oh Nein, ich hab mir beim Einparken den Kotflügel zerkratzt.
12:38
Dein Fahrlehrer wäre sicher stolz auf dich.
12:42
Nachricht schr...

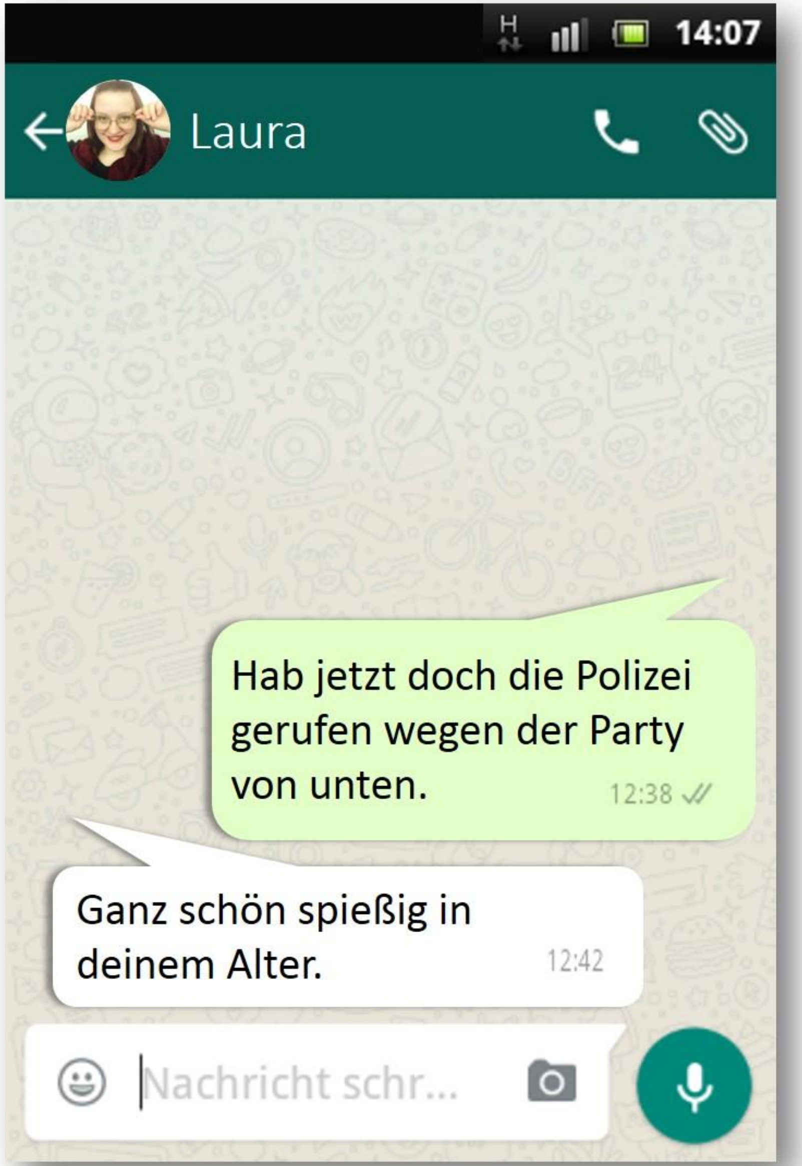
14:07
Laura
Hab jetzt doch die Polizei gerufen wegen der Party von unten.
12:38
Ganz schön spießig in deinem Alter.
12:42
Nachricht schr...

16

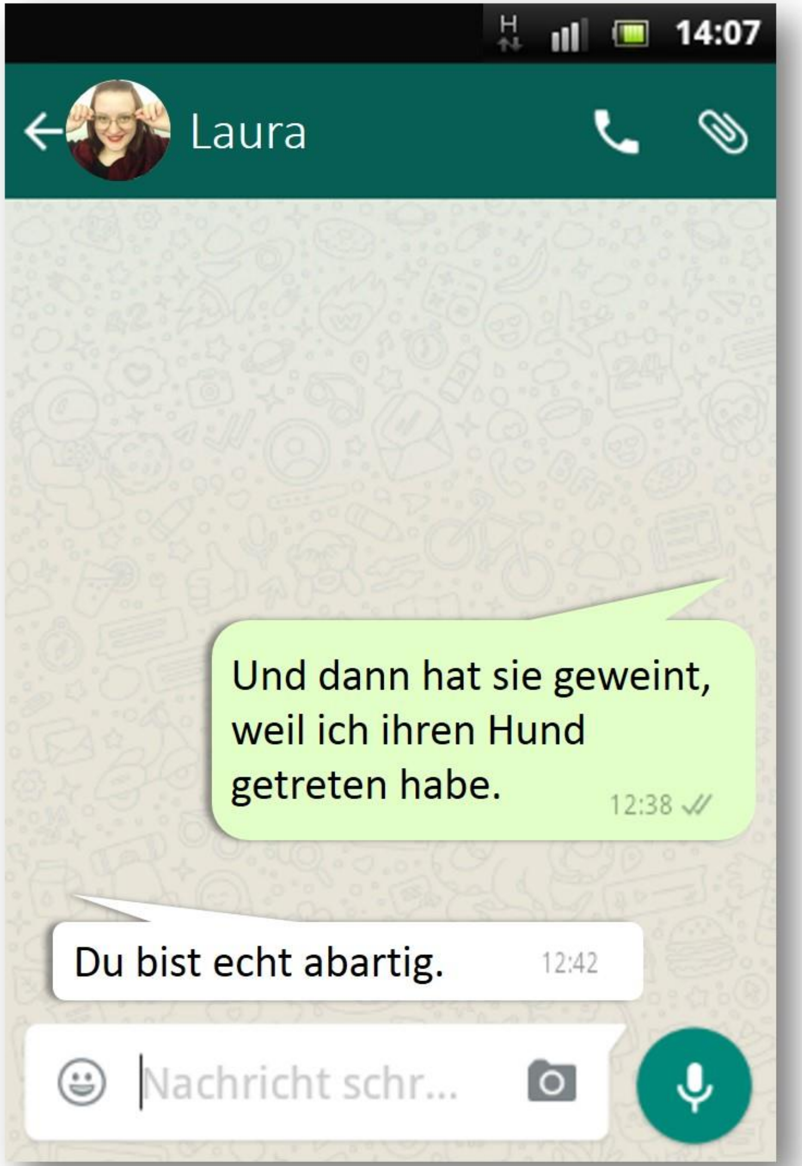
14:07
Laura
Und dann hat sie geweint, weil ich ihren Hund getreten habe.
12:38
Du bist echt abartig.
12:42
Nachricht schr...

17

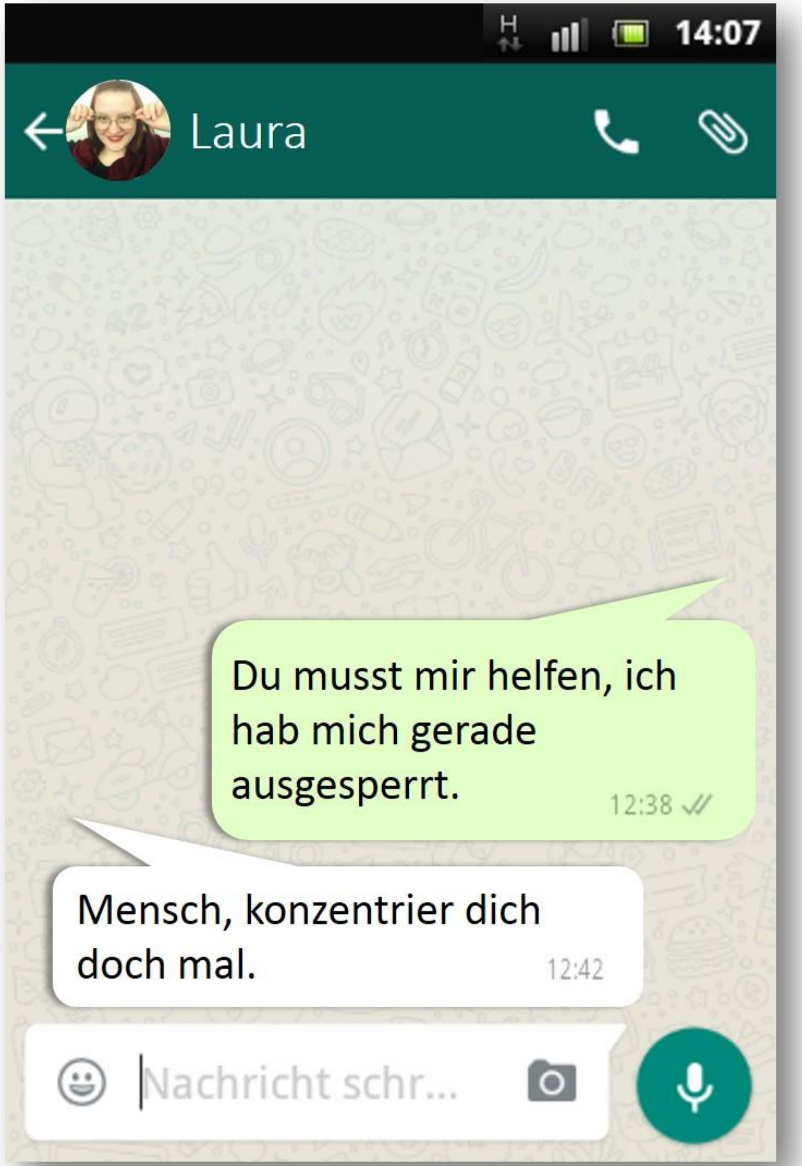
H
14:07
Laura
Du musst mir helfen, ich hab mich gerade ausgesperrt.
12:38
Mensch, konzentrier dich doch mal.
12:42
Nachricht schr...

18

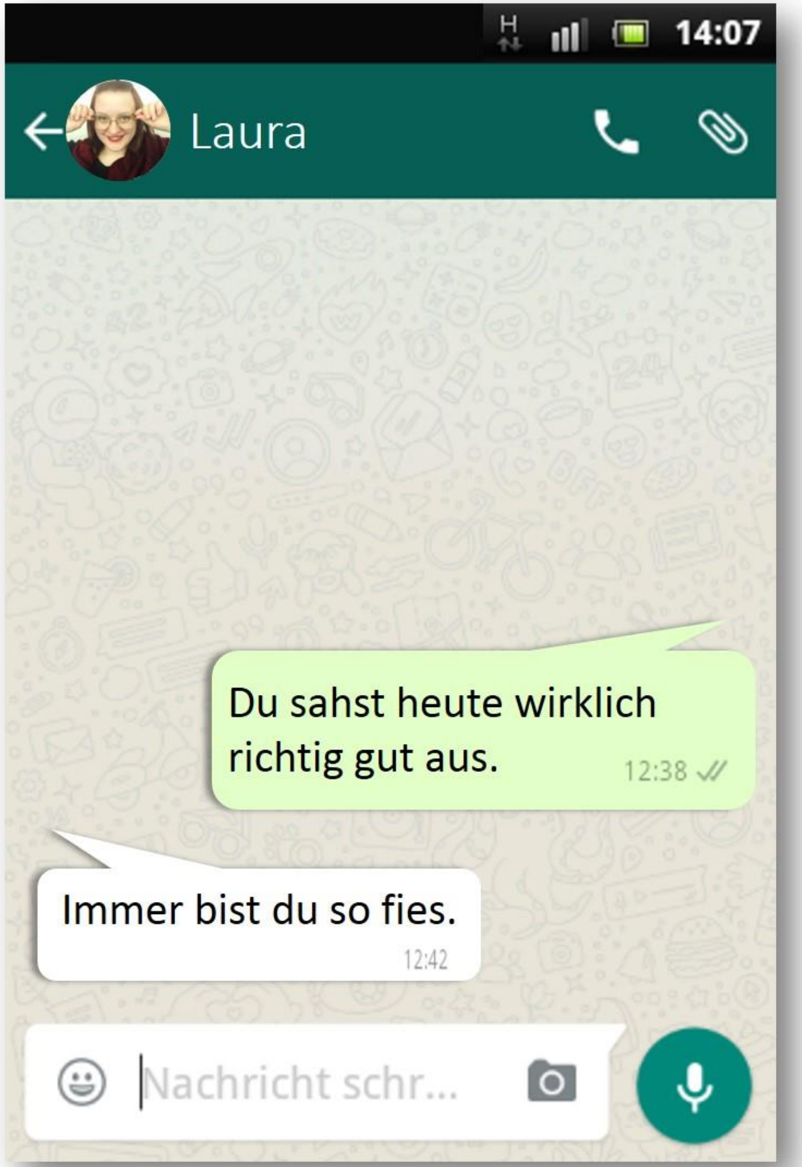
14:07
Laura
Du sahst heute wirklich richtig gut aus.
12:38
Immer bist du so fies.
12:42
Nachricht schr...

19

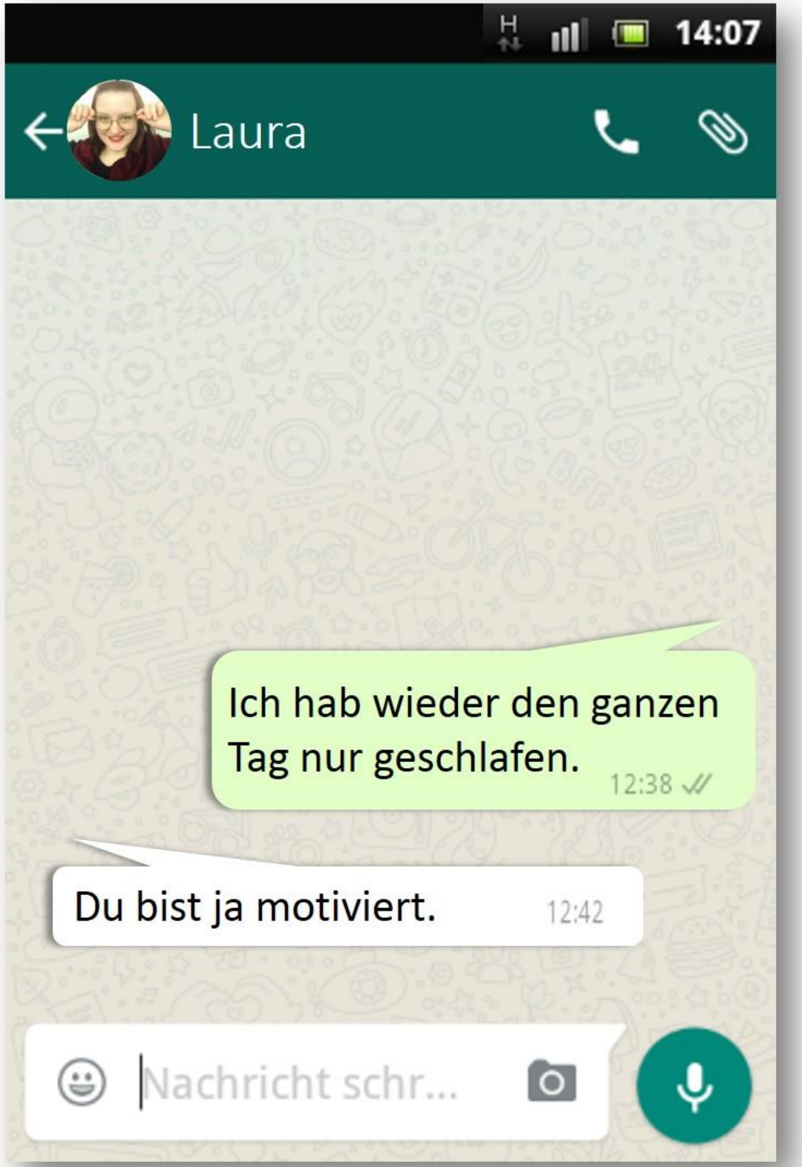
14:07
Laura
Ich hab wieder den ganzen Tag nur geschlafen.
12:38
Du bist ja motiviert.
12:42
Nachricht schr...

20

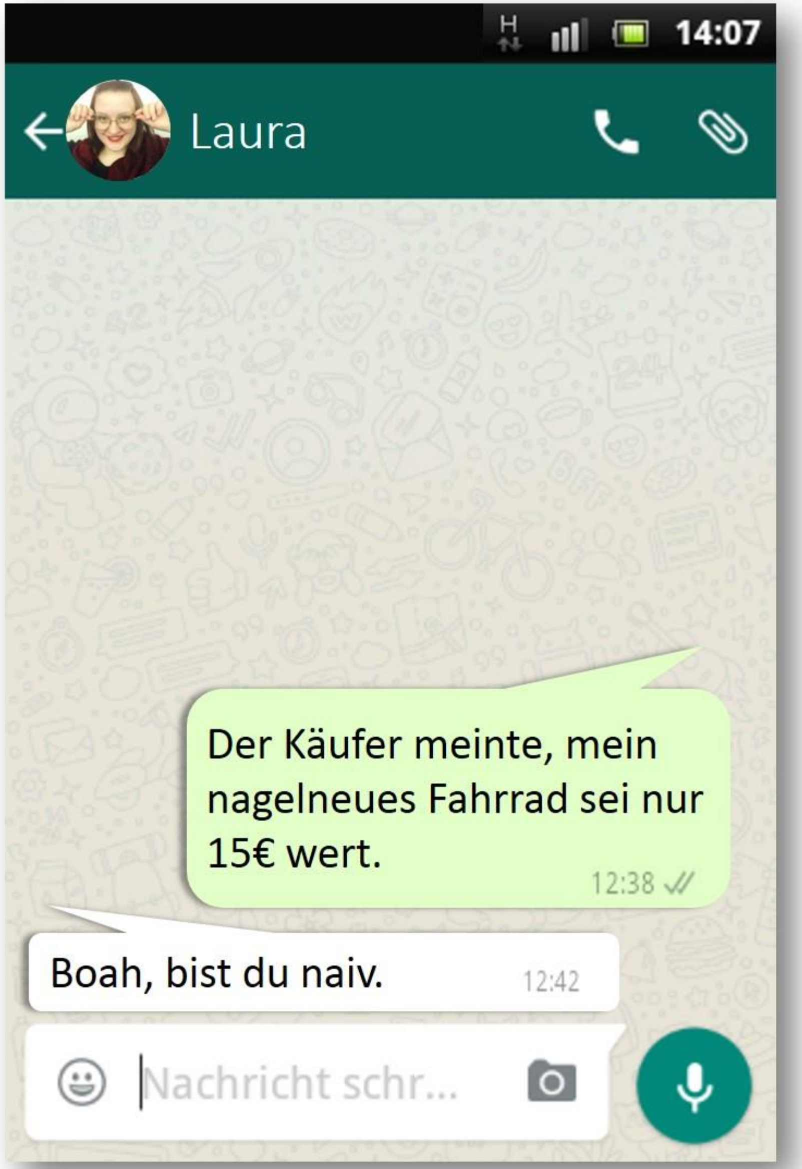

14:07
Laura
Der Käufer meinte, mein nagelneues Fahrrad sei nur 15€ wert.
12:38
Boah, bist du naiv.
12:42
Nachricht schr...
